# Flexible uncertainty calibration for machine-learned interatomic potentials

**DOI:** 10.1038/s41524-026-02080-3

**Published:** 2026-04-27

**Authors:** Cheuk Hin Ho, Christoph Ortner, YangShuai Wang

**Affiliations:** 1 Department of Mathematics, University of British Columbia, Vancouver, BC Canada; 2 Department of Mathematics, National University of Singapore, Singapore, Singapore

**Keywords:** Materials science, Mathematics and computing, Physics

## Abstract

Reliable uncertainty quantification (UQ) is essential for developing machine-learned interatomic potentials (MLIPs) in predictive atomistic simulations. Conformal prediction (CP) is a statistical framework that constructs prediction intervals with guaranteed coverage under minimal assumptions, making it an attractive tool for UQ. However, existing CP techniques, while offering formal coverage guarantees, often lack accuracy, scalability, and adaptability to the complexity of atomic environments. In this work, we present a flexible uncertainty calibration framework for MLIPs, inspired by CP but reformulated as a parameterized optimization problem. This formulation enables the direct learning of environment-dependent quantile functions, producing sharper and more adaptive predictive intervals at negligible computational cost. Using the foundation model MACE-MP-0 as a representative case, we demonstrate the framework across diverse benchmarks, including ionic crystals, catalytic surfaces, and molecular systems. Our results achieve substantial improvements in uncertainty-error correlation, improve the detection of high-error configurations for active learning, and transfer reliably across distinct exchange-correlation functionals. Importantly, it is general, data efficient, and compatible with diverse MLIP architectures and baseline UQ schemes, offering a practical route toward robust and transferable atomistic simulations.

## Introduction

Machine-learned interatomic potentials (MLIPs) have emerged as a powerful alternative to traditional empirical force fields and first-principles methods, enabling large-scale and high-throughput atomistic simulations with near DFT accuracy at a fraction of the computational cost^1–11^. They have been applied successfully in diverse areas such as materials discovery and molecular dynamics (MD). A central challenge, however, is to ensure the reliability of MLIP predictions when applied beyond their training distribution. This concern has motivated increasing interest in uncertainty quantification (UQ) as a means to assess predictive reliability and to guide robust atomistic simulations^12–16^.

A wide range of UQ strategies has been explored. Ensemble methods estimate predictive uncertainty from the variance of quantities of interest among models trained with different data splits^17,18^, potential forms^19,20^, or initializations^21,22^. Distance-based methods quantify dissimilarity between new configurations and the training set using measures such as D-optimality^23^, atomic fingerprints^24^, or latent space distances^25^. Probabilistic frameworks, including dropout-based Bayesian inference^26^ and a posteriori adaptivity^27,28^, also provide uncertainty estimates. These approaches have proven highly effective in driving active learning^29,30^, guiding transferability screening^31^, and assessing simulation stability in long MD trajectories^32,33^. However, the uncertainty estimates derived from these methods are often heuristic scores such as ensemble variance or latent space distances, rather than statistically calibrated probabilities. Consequently, while they successfully rank configurations, their absolute magnitudes may not directly correspond to the true error, and they lack formal coverage guarantees. Bridging the gap between these informative heuristic scores and statistically rigorous prediction intervals remains a key opportunity for improving the reliability and interpretability of atomistic simulations.

Conformal prediction (CP) provides a promising direction. It is a model-agnostic statistical framework that constructs prediction intervals with finite-sample coverage guarantees under minimal assumptions^34–36^. Recent works have applied CP in atomistic modeling^29,37,38^, allowing calibration of errors to actual physical units. Still, existing implementations face two major drawbacks. First, the training and calibration procedures are decoupled, which reduces both accuracy and efficiency. Second, the expressiveness of the method is limited, making it difficult to adapt prediction intervals to the complexity of local atomic environments.

To address these challenges, we introduce a flexible calibration framework in which an analog of the conformal quantile is learned as a smooth function of the local atomic environment rather than fixed as a global scalar. This enables predictive intervals to vary across space and adapt to complex structural features, improving both coverage and sharpness. Formally, the scalar quantile in the regular CP objective is replaced by a parameterized function trained with the pinball loss over the atoms in the calibration set. For robustness when nominal uncertainties are small, we further align the predicted interval directly to the reference force error using a physically motivated weight that emphasizes large deviations. The resulting objective is interpretable, numerically stable, and compatible with a wide range of MLIP architectures.

**Fig. 1:**
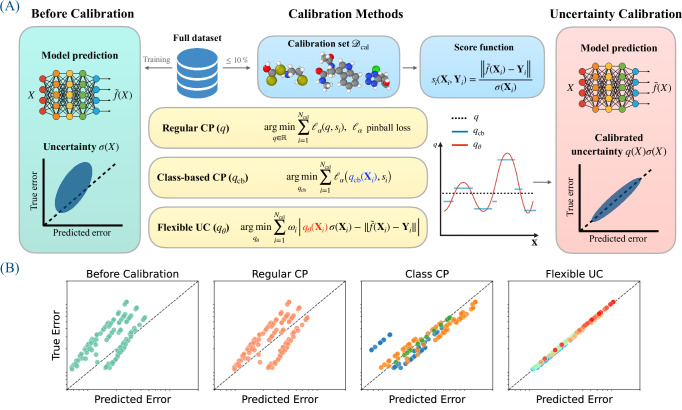
Conceptual illustration of the uncertainty calibration framework. **A** Workflow and calibration schemes. **B** Representative comparison of calibration outcomes. Before calibration and regular CP show systematic misalignment between predicted and true errors. Class CP improves alignment by grouping atomic environments into discrete classes, shown in different colors. Flexible UC further refines this by learning a smooth, environment-dependent mapping.

The design is conceptually related to class-based CP^39^ but enables fully data-driven and continuous adaptation within modern MLIP pipelines. Applied to the MACE-MP-0 foundation model with the LLPR baseline method^40^, the framework yields substantial improvements over standard CP in uncertainty calibration on various benchmarks, including ionic crystals, catalytic surfaces, and molecular datasets. It provides adaptive, site-resolved confidence measures that closely track true errors and improve the selection of high-error configurations for active fine-tuning and MD. The method introduces negligible computational overhead relative to the evaluation of the baseline model, remains data efficient with only a modest number of calibration configurations, and reliably transfers across distinct exchange-correlation (XC) functionals. Together, these properties define a principled and broadly applicable framework for uncertainty calibration in MLIPs. The approach is general, data efficient, and compatible with a wide range of architectures and baseline UQ schemes, supporting robust and scalable atomistic simulations. A schematic illustration of the proposed uncertainty calibration framework and its workflow is provided in Fig. 1.

This paper is organized as follows. “Methods” introduces the regular CP framework, outlines its limitations for machine-learned interatomic potentials, and presents the class-based CP and the flexible calibration approach. “Results” reports numerical experiments across diverse datasets, demonstrating improved calibration accuracy, data efficiency, and generalization. “Discussion” concludes with a summary of the main findings and discusses directions for future work. Additional details and supplementary numerical results are provided in the Supplementary Information (SI).

## Results

To demonstrate the effectiveness of the proposed uncertainty calibration framework, we conduct a series of numerical experiments based on the state-of-the-art MACE architecture^4^, which enables us to undertake a broad range of tests within a single framework. MACE extends the Atomic Cluster Expansion (ACE)^41^ by incorporating higher-order equivariant message passing through tensor products (see SI Section 2 for details). We employ the recently released foundation model MACE-MP-0b3^42^, trained on the MPtraj dataset, together with LLPR-based uncertainty estimates^40^ as the baseline (see SI Section 3 for background).

Our evaluation proceeds in four stages, each addressing a different aspect of calibration. (1) We begin by examining baseline performance on both task-specific and general-purpose datasets, and by assessing the computational efficiency of the approach (“Uncertainty calibration with atomistic foundation model”). (2) We then move to a more challenging setting, testing how well the method generalizes to unseen atomic environments in catalytic reaction pathways (“Generalization to unseen atomic environments: catalytic reaction pathways” (3) Next, we demonstrate how calibrated uncertainties can guide fine-tuning in MD simulations, highlighting their value for practical workflows (“Uncertainty-driven identification of high-error configurations in MD”). (4) Finally, we test whether calibrated uncertainties can be transferred across different XC functionals, an important aspect of multi-fidelity and multi-functional training (“Uncertainty transfer across exchange–correlation functionals”).

In each case study, we compare three calibration strategies. Regular CP applies a single global quantile, obtained by solving Eq. (3) with *α* = 0.5. We select this value to target the conditional median, providing a robust measure of typical uncertainty that is less sensitive to outliers than extreme quantiles, which balance over/under confidence for calibrating model uncertainty to actual errors. Class-based CP partitions local atomic environments into discrete groups. We typically set *N*_class_ = 20 if there is no further explicit mentioned and we construct the partition using a Gaussian mixture model^43^. For datasets with moderately diverse environments (cf. “Uncertainty transfer across exchange–correlation functionals”), the number of classes is empirically increased until the score distributions are well separated, beyond which further refinement has little effect. Flexible UC, our proposed approach, employs a feed-forward neural network that takes MACE descriptors as input and outputs a multiplicative scaling factor applied to the baseline estimate *σ*. Full implementation details are given in SI Section 4.6.

### Uncertainty calibration with an atomistic foundation model

We first evaluate and calibrate uncertainties predicted by the pretrained atomistic foundation model MACE-MP-0b3 using the LLPR baseline. No additional training or fine-tuning is performed; our focus here is solely on post hoc calibration of the existing model predictions.

As a starting point, we begin with the LiCl dataset from ref. ^44^, a prototypical ionic compound with simple composition and well-separated local atomic environments. Its moderate size and distinct structural motifs make it an ideal test case for evaluating the initial performance of UQ methods. Beyond this case-specific benchmark, we also include evaluations on larger public datasets such as MPtraj^45^ and MATPES^46^ to demonstrate the broader applicability of our method.

#### Calibration

**Fig. 2:**
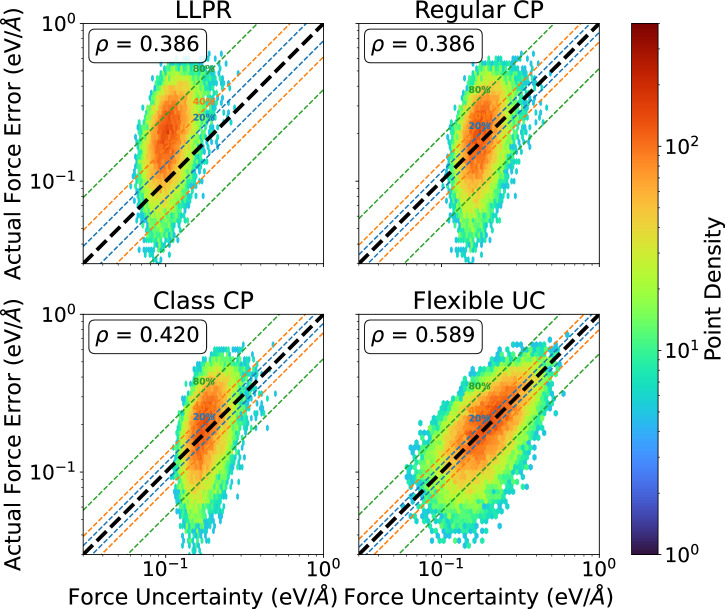
Comparison of uncertainties from LLPR, regular CP, class-based CP, and flexible UC on LiCl dataset. *ρ* denotes Spearman's rank correlation coefficient.

10% of configurations are drawn randomly (uniform) from the dataset to form the calibration set. Fig. 2 presents a comparison of predicted force uncertainties versus actual force errors across four uncertainty estimation/calibration schemes: LLPR (without CP), regular CP, class-based CP, and our proposed flexible uncertainty calibration. To quantify the alignment between predicted uncertainty and empirical error, we report the Spearman rank correlation coefficient *ρ* (see SI Section 1.1 for definition), which is widely used to assess monotonic relationships in UQ^47^.

The results indicate that regular CP offers limited calibration performance, reflected by the identical Spearman coefficients (*ρ* = 0.386) for the LLPR baseline and CP. The associated error-uncertainty distributions only show a difference in scale. The class-based CP yields a modest improvement by incorporating four empirically defined atomic classes, leading to a slight quantitative improvement to *ρ* = 0.420. In contrast, our proposed flexible calibration framework qualitatively improves the monotonic alignment between predicted uncertainties and actual errors, and achieves a markedly higher Spearman coefficient of *ρ* = 0.589, representing a 53% improved correlation as measured by the Spearman coefficient over the baseline LLPR method.

These results reveal a key limitation of regular CP in atomistic systems^38^: while global rescaling adjusts uncertainty magnitudes, it fails to improve their alignment with actual errors. In contrast, our flexible, data-driven calibration better captures local error patterns. To assess broader applicability, we provide additional results on the MPtraj and MATPES datasets in SI Section 4.2. We acknowledge, however, that the effectiveness of Flexible UC depends on the nature of the error distribution. The method is powerful when prediction errors are systematic and strongly correlated with local atomic structure, as the calibration model can effectively leverage descriptors to learn a precise rescaling factor for these specific local environments. This likely accounts for the substantial performance gains in LiCl, Catalysis and OMOL dataset (see following sections). In contrast, when errors are with less structure correlation and limits in data distribution shits, as in MPtraj and MATPES, they would naturally limit the calibration improvement.

#### **Efficiency**

An essential requirement for a practical uncertainty calibration method is that it should not introduce significant computational overhead relative to baseline model evaluation (i.e., model prediction and LLPR uncertainty estimation in this work). To assess efficiency, we benchmarked the evaluation time of the calibrated quantile ${\widehat{q}}_{\theta }$ when applied alongside the MACE-LLPR model on the LiCl dataset. The results, summarized in Table 1, show that ${\widehat{q}}_{\theta }$ contributes only 0.02% of the total evaluation time. While the absolute cost of the LLPR baseline may vary depending on implementation optimization, the computational cost of Flexible UC consists solely of a lightweight MLP forward pass. This ensures that calibration remains negligible in cost relative to a typical MLIP evaluation. This demonstrates that the proposed method delivers reliable uncertainty estimates at virtually no extra computational expense, ensuring its suitability for large-scale MD simulations and active learning workflows.

**Table 1:** Evaluation time (in seconds) of different calibration methods on the LiCl benchmark

Method	Evaluation time (s)
MACE	0.0182 (±0.0339)
MACE-LLPR	0.9766 (±0.0516)
Regular/class CP	0.0001 (±0.0001)
Flexible UC	0.0002 (±0.0001)

The timings are obtained by evaluating the corresponding architecture on the whole training set. Both class-based CP and the proposed flexible UC introduce negligible overhead compared with baseline MACE-LLPR evaluation or a standard MACE evaluation. This also demonstrates the need for a more efficient baseline uncertainty estimate.

### Generalization to unseen atomic environments: catalytic reaction pathways

To assess generalization to configurations not present in the calibration set, we evaluate its performance on a catalytic reaction pathway dataset^48^. This dataset includes both non-doped and Pt-doped catalytic surfaces. Note that while the MACE foundation model has encountered Pt species in its pre-training data (e.g., in bulk or molecular forms), both the foundation model and the quantile function have not been exposed to these specific Pt-doped surface configurations. Thus, this test assesses the method’s robustness to shifts in the local structural environment rather than to entirely unknown chemical elements. Specifically, the model is calibrated on non-doped surfaces and used to estimate uncertainties on Pt-doped configurations.

We hypothesize that our method can generalize across such shifts due to the shared chemical descriptors (e.g., atomic species) embedded in the MACE representation. Crucially, however, while the foundation model provides robust features, it is the Flexible UC framework that effectively leverages them to predict OOD errors.

**Fig. 3:**
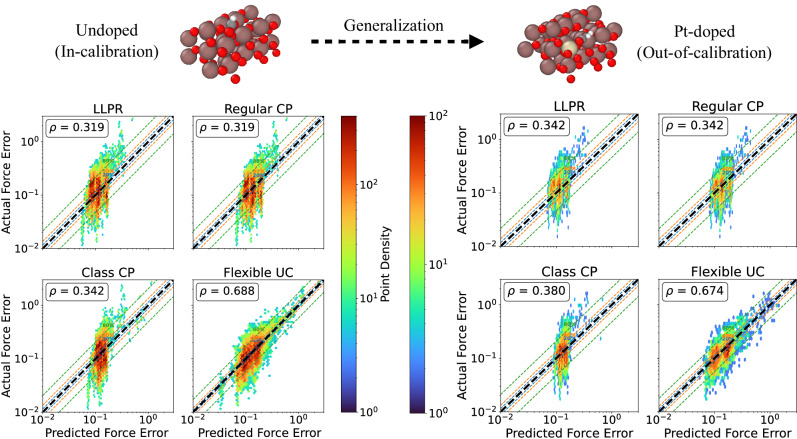
Uncertainty estimates from LLPR, regular CP, class CP, and flexible UC on catalytic surface data. Left: undoped configurations (in-calibration). Right: Pt-doped configurations (out-of-calibration). Regular and class-based CP show similar behavior in both cases, whereas flexible UC substantially improves generalization to unseen Pt-doped environments. All force units are reported in eV/Å.

We begin by evaluating the calibration performance on non-doped catalytic surfaces with only 5% of the available data. We use five classes for class CP. As shown in Fig. 3, both regular CP and class-based CP yield limited improvements: regular CP leaves the error-uncertainty correlation largely unchanged, while class-based CP leads to only a modest increase in the Spearman rank coefficient. In contrast, the flexible uncertainty calibration method achieves much better alignment between predicted uncertainties and actual errors, reflected by a Pearson correlation coefficient of 0.688. These results are consistent with our observations on the LiCl dataset (cf. “Uncertainty calibration with atomistic foundation model”).

To assess generalization to previously unseen atomic environments, we evaluate the calibrated uncertainties on Pt-doped surfaces. Notably, the model was never exposed to these Pt-containing configurations during calibration. Despite this, the flexible method maintains strong performance, with the Pearson coefficient increasing from 0.347 (before calibration) to 0.677 (after calibration), and a visibly tighter error-uncertainty scatter. These results suggest that our approach is not heavily dependent on the specific choice of calibration set ${{\mathcal{D}}}_{{\rm{cal}}}$, and can generalize robustly across varying chemical compositions. While this generalization benefits from the robust representations of the foundation model, the substantial improvement over the baseline demonstrates that our calibration is essential to effectively translate these features into reliable error estimates.

**Fig. 4:**
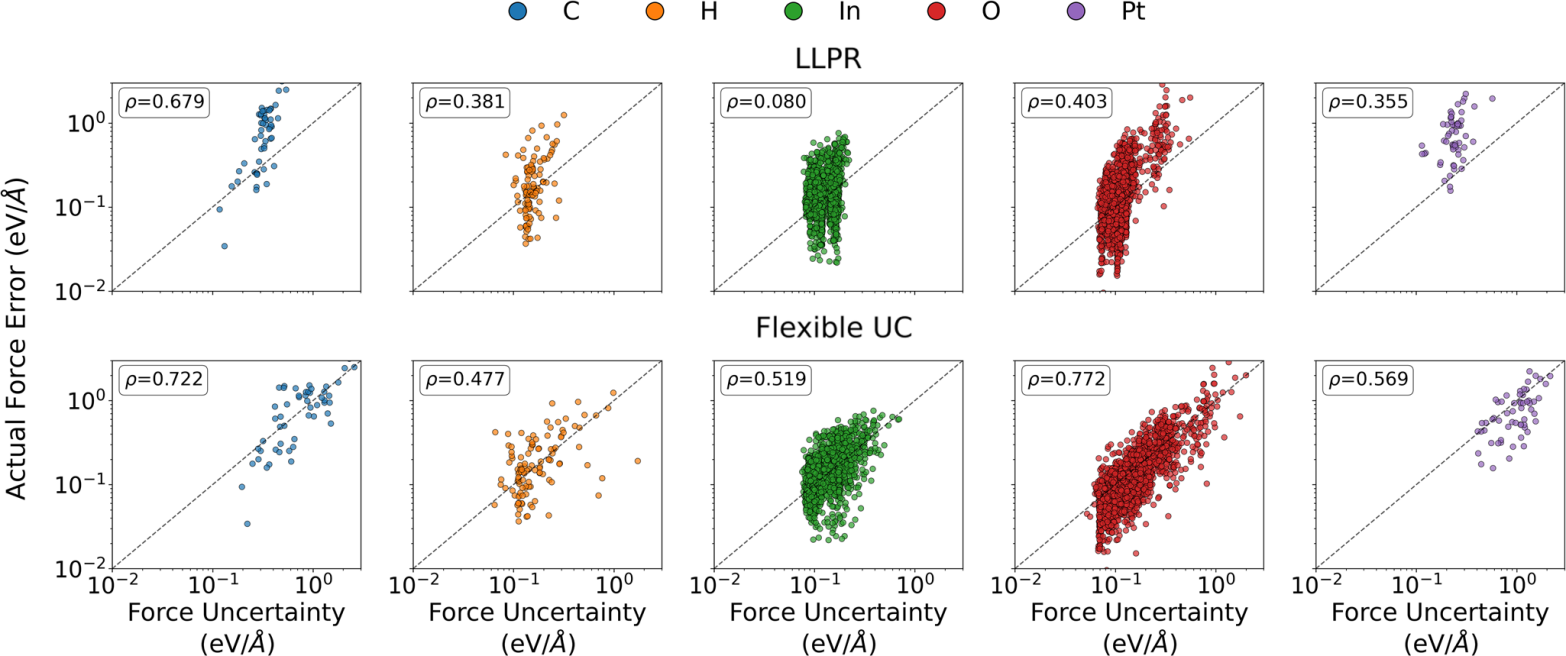
Species-resolved calibration of force uncertainties on Pt-doped surfaces. Predicted force uncertainties versus actual errors are shown for different elements. Top: regular conformal prediction. Bottom: flexible calibration. Although Pt atoms are excluded from the calibration set, the proposed method yields improved correlation (*ρ*) across all species.

To further examine the generalization performance, Fig. 4 presents species-resolved scatter plots of predicted force errors versus calibrated uncertainties. The top row corresponds to LLPR (before calibration), and the bottom row to the flexible uncertainty calibration method. Notably, Pt atoms are absent from the calibration set. As shown, all species exhibit improved alignment between error and uncertainty after calibration. In particular, the flexible method generalizes effectively to unseen atomic environments, yielding improved correlation for Pt atoms. This demonstrates the robustness of the method to extrapolation beyond the calibration distribution.

**Fig. 5:**
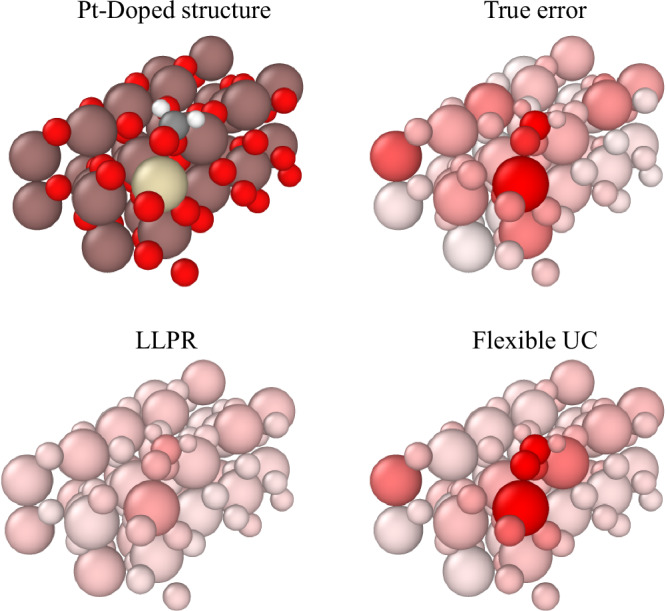
Uncertainty estimates from LLPR and flexible UC, together with true force errors, for a representative Pt-doped surface configuration (atomic structure shown on the left). Errors and uncertainties are assigned on a per-atom basis, with darker colors indicating atoms exhibiting larger values.

To further highlight the advantages of flexible calibration, Fig. 5 compares per-atom predicted uncertainties with reference force errors for a representative Pt-doped configuration. Flexible UC yields sharper and more accurate localization of high-error atoms, closely matching the true error distribution. In contrast, LLPR uncertainties are diffuse and show weak correlation with the actual errors. This improvement is particularly important for large-scale simulations, where direct DFT validation is infeasible for detecting erroneous sites. Crucially, the quantitative assessment in Table 2 reveals a limitation of LLPR: its identification accuracy for high-error configurations drops significantly (as low as ~14%). This high failure rate could possibly be attributed to a systematic bias where LLPR’s uncertainty estimates consistently fail to flag the most informative samples. Using LLPR without calibration may therefore result in ineffective identification of high-force error sites. By calibration on existing LLPR uncertainty, flexible UC establishes a practical foundation for more effective active learning workflow by providing more reliable alignment between errors and uncertainties, as illustrated in the next section.

**Table 2:** Identification accuracy of the most erroneous configurations before (LLPR) and after calibration (Flexible UC) across different window sizes over the testing trajectory

Window size	LLPR	Flexible UC
40 ps	30.8%	69.2%
60 ps	33.3%	44.4%
80 ps	14.3%	57.1%
100 ps	20.0%	60.0%

Accuracy is defined as the percentage of time windows where the configuration with the maximum predicted uncertainty matches the configuration with the maximum true error.

### Uncertainty-driven identification of high-error configurations in MD

To demonstrate the practical impact of uncertainty calibration, we investigate its role in active fine-tuning within MD simulations. This requires the ability to identify high-error configurations, since they continuously generate new atomic configurations whose predictive errors are a priori unknown^32^. Without reliable uncertainty ranking, the batched selection^29,49^ risks populating the selection pool with low-error structures (as demonstrated in Table 2), leading to possible inefficient model refinement. While this limitation is well recognized in the broader active learning literature^22,32,50^, it has not, to our knowledge, been systematically examined in the context of fine-tuning MLIP foundation models.

To evaluate the capability of identifying high-error configurations, we initiate an NVT trajectory from a randomly selected configuration in the catalyst dataset described in the previous section. We sample uniformly from the first 20% of the trajectory to form a calibration set, train a flexible uncertainty calibration model on this subset, and then assess performance on the remaining 80% of the trajectory. This setup mimics a realistic active learning workflow, where calibration must be established on a limited portion of the data while robust uncertainty estimates are required for unseen configurations throughout the simulation.

We performed single-point DFT evaluations every 4 ps along the calibration trajectory, and a coarser 20 ps window on the testing trajectory to ensure the configurations are sufficiently decorrelated in each cases. Consecutive evaluations were grouped into segments, which we define as windows of a given size. Within each window, the configuration with the largest absolute force error relative to the DFT reference was identified as the most erroneous. The ability of each method to correctly detect these configurations on the testing trajectory is reported in Table 2. The results show that flexible UC consistently outperforms LLPR across all window sizes, reliably identifying high-error configurations that LLPR often fails to capture. Notably, even for larger windows where the increased number of configurations introduces additional uncertainty, flexible UC still achieves up to 50% accuracy, compared to 15–20% for LLPR.

**Fig. 6:**
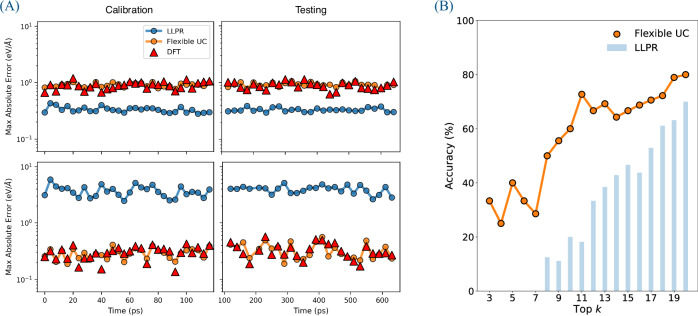
Uncertainty calibration improves error alignment and identification of high-error configuration selection. **A** Temporal evolution of predictive uncertainty and actual errors during an NVT simulation. Calibration and testing sets shown before (top) and after (bottom) fine-tuning. **B** Top-*k* accuracy for identifying structures with the highest force errors during molecular dynamics on the testing simulation. Orange markers show the Flexible UC method consistently outperforms the original LLPR uncertainties (blue bars) over different batch sizes, demonstrating improved calibration for high-error structure selection.

Beyond local window-based detection, we also assess the reliability of uncertainty estimates for batch selection. To quantify this, we evaluated the Top-*k* accuracy of the uncertainty estimates on the testing trajectory. We consider the top *k* configurations with the highest predicted uncertainty and calculate the fraction of these that correspond to true high DFT error configurations. As shown in Fig. 6B, the baseline LLPR yields, in general, result in lower precision across different batch sizes. In contrast, Flexible UC maintains a precision of ~40–50%. This confirms that while Flexible UC does not replace diversity-based sampling, it establishes the essential prerequisite for it: a high-quality candidate pool where uncertainty reliably signals error. Consequently, in active learning loops or iterative exploration, this ability to filter out false positives and pinpoint truly erroneous configurations ensures targeted data acquisition and efficient model refinement.

To further assess the impact of calibration on predictive uncertainties, we carried out additional NVT simulations initialized from the same structure. Configurations were sampled along the trajectory, followed by a single round of fine-tuning and subsequent uncertainty calibration using the proposed flexible UC framework. Figure 6A summarizes representative frames with reference DFT calculations before and after fine-tuning. Three key observations emerge: (i) prediction errors decrease substantially after fine-tuning; (ii) the original LLPR uncertainty, which previously underestimated the true error, becomes more conservative after fine-tuning; and (iii) once calibrated, the uncertainty aligns much more closely with the actual error on the testing trajectory, again confirming the effectiveness of the flexible UC method.

To the best of our knowledge, these findings demonstrate, for the first time, that large-scale atomistic foundation models can in principle be fine-tuned within an active learning workflow while maintaining reliable error detection. Although we illustrate only a single fine-tuning step, flexible UC establishes a solid basis for uncertainty-guided active learning. A complete fine-tuning scheme will be reported in future work, as the present study focuses on introducing and validating the flexible UC framework.

### Uncertainty transfer across XC functionals

A further advantage of the proposed framework lies in its ability to transfer calibrated uncertainty estimates across distinct data distributions, in particular across XC functionals. This capability is essential when constructing large-scale training sets that combine data generated from different functionals. To illustrate this point, we consider a model pre-trained on PBE and examine its transfer to alternative functionals such as PBEsol^51^ and *ω*B97M-V/def2-TZVPD^52^. For a given atomic index *i*, the relevant discrepancy can be expressed as

1 $$\left|{\widetilde{{\bf{F}}}}_{i}^{{\rm{PBE}}}-{{\bf{F}}}_{i}^{{\rm{XC}}}\right|=\left|{\widetilde{{\bf{F}}}}_{i}^{{\rm{PBE}}}-{{\bf{F}}}_{i}^{{\rm{PBE}}}-{\varepsilon }_{i}^{{\rm{corr}}}\right|,$$

where ${\widetilde{{\bf{F}}}}_{i}^{{\rm{PBE}}}$ denotes the force predicted by the PBE-pretrained MLIP, ${{\bf{F}}}_{i}^{{\rm{PBE}}}$ the corresponding DFT reference, and ${\varepsilon }_{i}^{{\rm{corr}}}$ the functional correction that accounts for systematic differences between XC approximations. Configurations of interest therefore, correspond either to regions where the PBE model error $| {\widetilde{{\bf{F}}}}_{i}^{{\rm{PBE}}}-{{\bf{F}}}_{i}^{{\rm{PBE}}}|$ is large, or where the cross-functional shift $| {\varepsilon }_{i}^{{\rm{corr}}}|$ is significant. It is important to note that while the energy difference between functionals often includes a constant shift, the force discrepancy arises from changes in the local curvature of the potential energy surface. Consequently, the errors between different functionals ${\varepsilon }_{i}^{{\rm{corr}}}$ depend on the local atomic environment and are not globally uniform. Under the mild assumption that cancellation between the two terms ${{\bf{F}}}_{i}^{{\rm{PBE}}}$ and ${\varepsilon }_{i}^{{\rm{corr}}}$ is negligible, maximizing (1) is equivalent to maximizing both or either one of the PBE model error and cross-functional shift. Our flexible calibration framework provides a principled and cost-efficient means of estimating (1) without exhaustive labeling.

This transfer setting is particularly important because uncertainty calibration is not only required to correct model confidence within a single functional, but also to maintain consistency across heterogeneous datasets that underpin emerging foundation models. By exploiting calibrated error estimates from the source functional and learning only a lightweight correction term from a small number of target-functional calculations, one can significantly reduce the labeling cost while retaining reliable uncertainty estimates. In practice, this enables systematic fine-tuning across functionals and supports the integration of diverse data sources into a unified MLIP framework, thereby extending the applicability of atomistic foundation models to broader chemical and physical domains. To the best of our knowledge, this constitutes the first systematic demonstration of uncertainty calibration for cross-functional transfer.

To demonstrate this transferability, we consider the MACE-MP-0b3 model with LLPR-based uncertainties. By construction, LLPR captures prediction errors relative to the PBE functional (consistent with the MPtraj training data), but it does not directly reflect discrepancies introduced by other XC functionals. In this context, our framework enables PBE-based uncertainty estimates to be transferred to the error measure in (1), thereby extending their applicability to different functionals. We evaluate this capability on two challenging benchmarks: the HEA25 dataset^53^, computed with the PBEsol functional^51^, and a subset of the Open Molecule 2025 dataset^52^, generated at the *ω*B97M-V/def2-TZVPD level of theory, which departs substantially from PBE.

#### **HEA25**

**Fig. 7:**
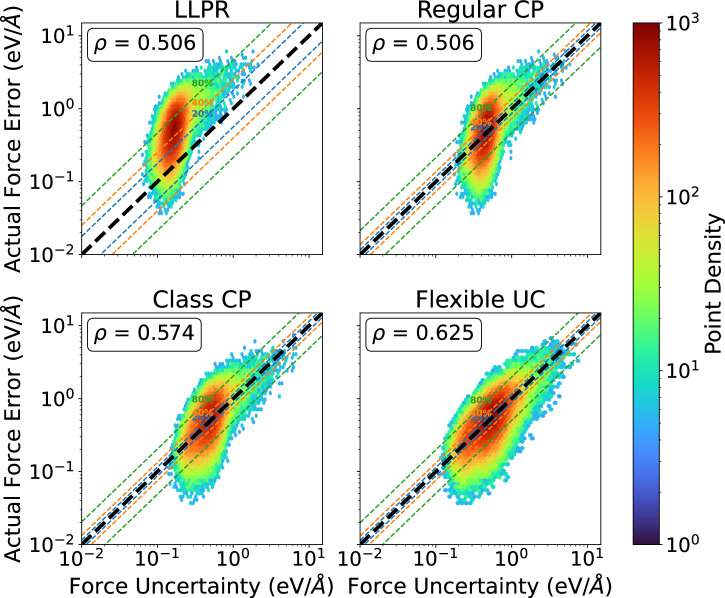
Calibration performance on the HEA25 dataset computed with the PBEsol functional, comparing uncertainty estimates from LLPR, regular CP, class-based CP, and flexible UC. Flexible UC provides the most significant improvement in correlating predicted uncertainties with actual errors.

Figure 7 reports calibration results on the HEA25 dataset, which targets chemically complex, high-entropy alloys and was computed with the PBEsol functional. Since PBEsol is relatively close to the PBE functional used for pre-training, both LLPR and regular CP already provide moderately reasonable uncertainty estimates. In particular, LLPR is able to capture high-error configurations (e.g., errors on the order of 1 eV/Å) with fair accuracy. Class-based CP provides a slight improvement by leveraging empirical partitioning of the input space, but the correlation gain remains limited. In contrast, the flexible calibration approach yields a marked improvement in aligning predicted uncertainties with actual errors, as reflected by the higher Spearman rank correlation coefficient (*ρ* = 0.625). This demonstrates that even for systems relatively close to the training distribution, flexible UC substantially enhances the reliability and robustness of uncertainty estimates.

#### **Open Molecule 2025**

**Fig. 8:**
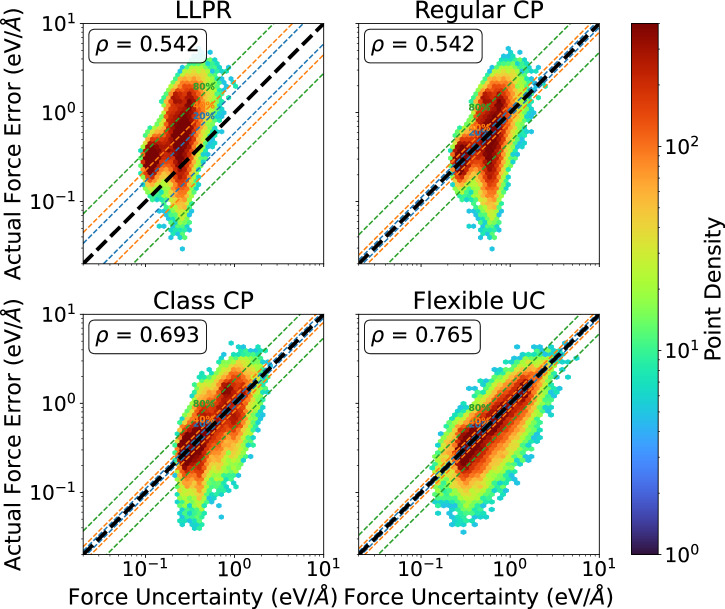
Predicted force uncertainties versus actual force errors on the Open Molecule 2025 dataset. Flexible UC achieves the strongest correlation between predicted uncertainties and observed errors under substantial distribution shifts.

**Fig. 9:**
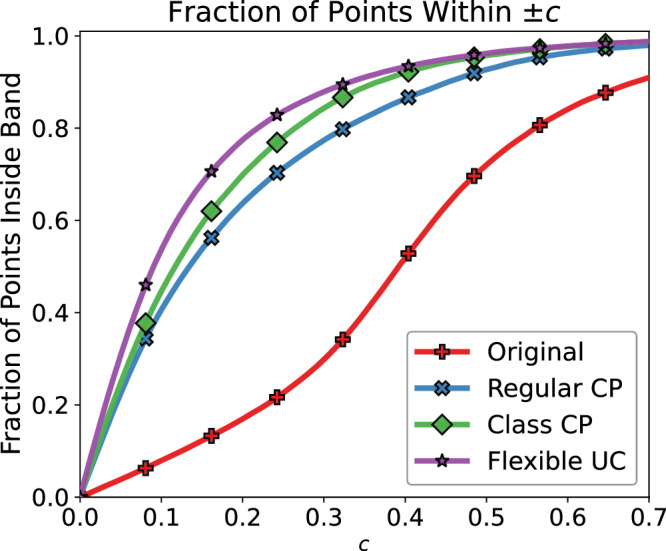
Calibration coverage on the Open Molecule 2025 dataset. The curves show the number of atomic forces lying within uncertainty intervals of varying width. Flexible UC provides the most reliable calibration compared to baseline methods.

To further evaluate transferability under substantial distribution shifts, we benchmark the proposed method on a subset of the Open Molecule 2025 dataset^52^, which differs significantly from MPtraj in both chemical composition and the XC functional. From the validation split, we randomly select 2000 charge-neutral molecular configurations, corresponding to roughly 90,000 atomic sites in total. The results are summarized in Fig. 8. In this case, LLPR-based uncertainties fail to capture the correlation between predicted and true errors, as most predictions collapse into a narrow uncertainty range. Regular CP provides no improvement beyond LLPR, while class-based CP achieves moderate gains, likely reflecting the greater separability of local atomic environments in molecular systems compared to extended solids. The proposed flexible UC framework again delivers the best correlation on this challenging benchmark. Furthermore, coverage analysis (Fig. 9) confirms that flexible UC consistently provides better-calibrated intervals, with a larger fraction of points falling within prescribed confidence bands.

These results demonstrate the robustness of flexible UC in handling strong distributional shifts, highlighting its potential as a general calibration strategy for atomistic foundation models.

#### **Calibration efficiency**

**Fig. 10:**
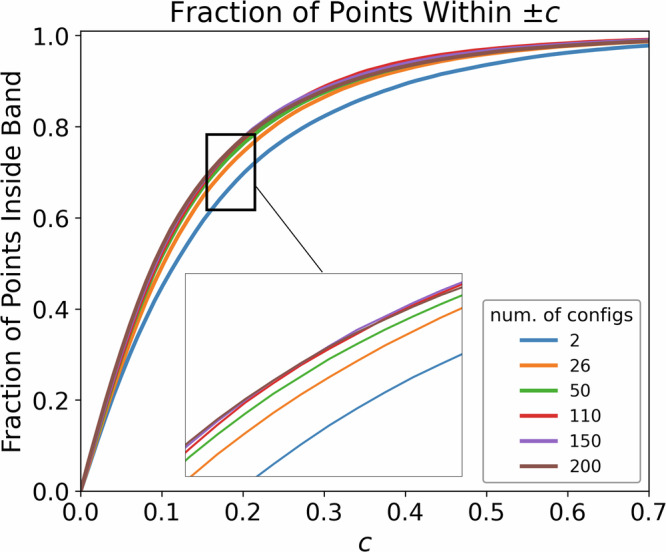
Convergence of the coverage curve on the Open Molecule 2025 dataset as a function of calibration set size. Reliable calibration is achieved with only a small number of configurations.

**Fig. 11:**
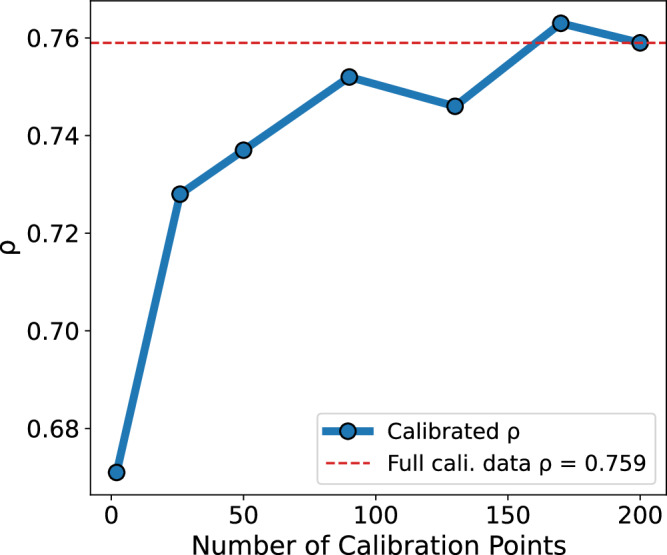
Convergence of the Pearson correlation coefficient on the Open Molecule 2025 dataset. Performance stabilizes after ~50 calibration configurations.

In our final test, we assess the efficiency of the flexible UC scheme by varying the number of calibration configurations. As shown in Figs. 10 and 11, both the coverage curve and the Pearson correlation coefficient improve rapidly with additional calibration data and plateau after only ~25 calibration configurations. This behavior reflects the fact that the effective calibration size scales with the total number of atoms rather than the number of configurations alone, thereby amplifying the information contained in each configuration. These results demonstrate that the proposed method is highly data efficient, making it practical even in scenarios where reference calculations are expensive and only limited calibration data are available.

## Discussion

We have presented a flexible uncertainty calibration framework for MLIPs, which reformulates CP into a learnable, environment-dependent quantile model. By embedding the quantile function into the predictive pipeline and optimizing it end-to-end, the method produces adaptive and well-calibrated uncertainty estimates that closely align with true errors. Applied to the MACE-MP-0 foundation model with LLPR, the framework yields significant improvements in calibration error while being extremely data efficient during training and incurring negligible computational overhead during evaluation. Extensive benchmarks across ionic crystals, catalytic surfaces, and molecular datasets demonstrate the method’s robustness, and practical tests show its value in active fine-tuning, MD, and cross-xc-functional transfer.

Looking ahead, several promising directions arise. First, extending the framework to jointly calibrate multiple physical quantities (energy, forces, stresses) could provide unified statistical guarantees across coupled observables. Second, leveraging site-resolved calibrated uncertainties for large-scale or long-time MD simulations would enable robust configuration screening and reliable uncertainty-guided active learning. Third, applying the method to data-scarce regimes such as phase transitions, chemical reactions, or battery interfaces could establish reliable UQ in scenarios where extrapolation risks are most severe. Taken together, this work establishes flexible uncertainty calibration as a practical and principled approach to improving the reliability of MLIPs and paves the way toward uncertainty-aware foundation models for atomistic simulation.

## Methods

In this section, we present new methods for calibrating heuristic uncertainty estimates in MLIP predictions. We begin by introducing regular CP to establish notation and background. We then review the generalization of the framework to class-based CP, designed to mitigate covariate shifts in testing distributions. We then propose a new uncertainty calibration approach, inspired by but not strictly within the framework of CP, and demonstrate its application to MLIPs in the context of calibrating force uncertainty.

### Conformal prediction

CP^34^ is a distribution-free and model-agnostic framework that calibrates uncertainty with guaranteed coverage under minimal assumptions.

To begin with, let $(\Omega ,{\mathcal{F}},{\mathbb{P}})$ be a probability space. We consider a data-generating process governed by a (unknown) joint distribution *P* on ${\mathcal{X}}\times {\mathcal{Y}}$, where ${\mathcal{X}}$ and ${\mathcal{Y}}$ denote the input and output spaces, respectively. We assume that ${\mathcal{Y}}$ is equipped with a norm ∥ ⋅ ∥ (possibly the Euclidean norm). Let ${{\mathcal{D}}}_{{\rm{train}}}={({{\bf{X}}}_{i},{{\bf{Y}}}_{i})}_{i=1}^{{N}_{{\rm{train}}}}$ be a training dataset consisting of *N*_train_ i.i.d. samples drawn from *P*.

Given ${{\mathcal{D}}}_{{\rm{train}}}$, we train a predictive model $\widetilde{f}:{\mathcal{X}}\to {\mathcal{Y}}$ with an associated heuristic uncertainty estimate $\sigma :{\mathcal{X}}\to {\mathbb{R}} > 0$. Practically, the uncertainty estimate *σ*(**X**) may be derived from ensemble methods, Monte Carlo dropout, Bayesian posterior variances, or other approximate epistemic UQ schemes^13,18,54,55^.

In many cases, *σ*(**X**) is inaccurate and requires calibration on a (finite) calibration set. Such a calibration set is analogously assumed to be comprised of i.i.d. samples from *P*, with an additional mild exchangeability assumption with test inputs^56^. Exchangeability means that the set of random variables ${{\mathcal{D}}}_{{\rm{cali}}}\cup \{{{\bf{X}}}_{{\rm{new}}}\}$ is permutation invariant for any testing configuration **X**_new_.

To proceed with calibrating *σ*(**X**), we define a score function $s:{\mathcal{X}}\times {\mathcal{Y}}\to {{\mathbb{R}}}_{\ge 0}$,

2 $$s({\bf{X}},{\bf{Y}}):=\frac{\left\Vert \widetilde{f}({\bf{X}})-{\bf{Y}}\right\Vert }{\sigma ({\bf{X}})},$$

which measures the discrepancy between the model prediction and the observed ground truth, normalized by the heuristic uncertainty estimate.

Using Eq. (2), calibration scores ${\left\{{s}_{i}:=s({{\bf{X}}}_{i},{{\bf{Y}}}_{i})\right\}}_{i=1}^{{N}_{{\rm{cal}}}}$ are computed for all samples in ${{\mathcal{D}}}_{{\rm{cal}}}$. Given a fixed *α* ∈ (0, 1), setting

3 $$\widehat{q}:={\rm{quantile}}\left({\{{s}_{i}\}}_{i=1}^{{N}_{{\rm{cal}}}},\frac{\lceil ({N}_{{\rm{cal}}}+1)(1-\alpha )\rceil }{{N}_{{\rm{cal}}}}\right)$$

ensures that

4 $$\begin{array}{rcl}1-\alpha & \le & {\mathbb{P}}\left(\parallel {{\bf{Y}}}_{{\rm{new}}}-\widetilde{f}({{\bf{X}}}_{{\rm{new}}})\parallel \le \widehat{q}\sigma ({{\bf{X}}}_{{\rm{new}}})\right)\\ & \le & 1-\alpha +\frac{1}{{N}_{{\rm{cal}}}+1}.\end{array}$$

Such a formulation, based on the leave-one-out augmented dataset^57^, ensures the marginal coverage guarantee (4). The scaled uncertainty $\widehat{q}\sigma ({{\bf{X}}}_{{\rm{new}}})$ can be regarded as the calibrated uncertainty. See SI Section 1.4 for further details and its Bayesian interpretation.

A key property that serves as the main motivation of our proposed framework in “Flexible uncertainty calibration” is that regular CP can be framed as an optimization problem. As shown in ref. ^58^, the conformal quantile $\widehat{q}$ in (3) can be equivalently obtained by solving

5 $$\widehat{q}=\mathop{\arg \,\min }\limits_{q\in {\rm{{\mathbb{R}}}}}\mathop{\sum }\limits_{i=1}^{{N}_{\mathrm{cal}}}{\ell }_{\alpha }(q,{s}_{i}),$$

where *ℓ*_*α*_ is the pinball loss function defined by

6 $${\ell }_{\alpha }(q,S):=\left\{\begin{array}{lc}(1-\alpha )(S-q) & \mathrm{if}S\ge q,\\ \alpha (q-S) & \mathrm{if}S < q.\end{array}\right.$$

The hyperparameter *α* in (6) again governs the trade-off between underestimation and overestimation in the calibrated uncertainty, equivalent to the effect of *α* on the choice of quantile in (3).

### Class-based CP

The conformal quantile $\widehat{q}$, obtained as the solution to Eq. (5), serves as a global scaling factor for calibrating heuristic uncertainty estimates. Since $\widehat{q}$ is shared across all inputs, regardless of the “local” quality of the heuristic uncertainty *σ*(**X**), this fundamentally limits the method's calibration accuracy. Moreover, regular CP is highly sensitive to covariate shift, further degrading its performance in distributionally shifted settings^39^. These limitations highlight the need for input-dependent rescaling, where each test point **X** is assigned its own adjustment factor to better capture the heterogeneity of uncertainty across the input space.

A natural extension of regular CP is to allow the rescaling parameter to vary across different input classes $\xi \subseteq {\mathcal{X}}$ with ${\mathcal{X}}={\bigcup ^{^\circ }}_{\xi }\xi$ so that the calibration becomes more fine-grained. One could first train a classification model that induces such a finite partition *ξ* ∈ Ξ of the input space, and perform calibration on each class, respectively. More precisely, calibration scores ${\left\{{s}_{i}^{\xi }:=s({{\bf{X}}}_{i},{{\bf{Y}}}_{i})| {{\bf{X}}}_{i}\in \xi ,\xi \in \Xi \right\}}_{i=1}^{{N}_{\xi ,{\rm{cal}}}}$ are computed for all samples in ${{\mathcal{D}}}_{{\rm{cal}}}$. Now, fix *α* ∈ (0, 1), setting

7 $${\widehat{q}}_{\xi }:={\rm{quantile}}\left({\{{s}_{i}^{\xi }\}}_{i=1}^{{N}_{{\rm{cal}}}},\frac{\lceil ({N}_{{\rm{cal}}}^{\xi }+1)(1-\alpha )\rceil }{{N}_{{\rm{cal}}}^{\xi }}\right)$$

ensures that, as shown in refs. ^39,59^,

8 $$\begin{array}{rcl}1-\alpha & \le & {\rm{{\mathbb{P}}}}\left(\parallel {{\bf{Y}}}_{\mathrm{new}}-\widetilde{f}({{\bf{X}}}_{\mathrm{new}})\parallel \le {\widehat{q}}_{\xi }\sigma ({{\bf{X}}}_{\mathrm{new}})\right)\\ & \le & 1-\alpha +\frac{| \Xi | }{({N}_{\mathrm{cal}}^{\xi }+1)\cdot {\rm{{\mathbb{P}}}}({{\bf{X}}}_{\mathrm{new}}\in \,\xi )}.\end{array}$$

for any testing configuration **X**_new_ ∈ *ξ*.

This can be thought of as rescaling uncertainties with a step function (w.r.t. a finite partition of input space) that is constant within each input class, see SI Section 1 for details. Analogously to (5), we can write this as an optimization problem

9 $${\widehat{q}}_{\mathrm{cb}}:=\mathop{\arg \,\min }\limits_{{q}_{\mathrm{cb}}\in {{\mathscr{F}}}_{\mathrm{cb}}}\mathop{\sum }\limits_{i=1}^{{N}_{\mathrm{cal}}}{\ell }_{\alpha }\left({q}_{\mathrm{cb}}({{\bf{X}}}_{i}),{s}_{i}\right),$$

where the subscript cb is used to distinguish ${\widehat{q}}_{{\rm{cb}}}$ from the quantile obtained in SI Section 1. In here, ${{\mathscr{F}}}_{{\rm{cb}}}$ denotes the class of step functions over a finite collection of classes Ξ, defined as

$${{\mathscr{F}}}_{{\rm{cb}}}:=\left\{x\mapsto \mathop{\sum }\limits_{\xi \in \Xi }{q}_{\xi }\,{\bf{1}}\{x\in \Xi \}:{q}_{\xi }\in {\mathbb{R}},\,\forall \xi \in \Xi \right\},$$

and **1**{ ⋅ } denotes the indicator function. This is equivalent to performing regular CP separately on each class *ξ*, thereby yielding a piecewise-constant function ${\widehat{q}}_{{\rm{cb}}}({\bf{X}})$ with ${q}_{\xi }={\widehat{q}}_{\xi }$ that assigns a distinct quantile value to each class.

As shown in ref. ^39^, Corollary 1 (and briefly discussed in SI Section 1), CP with class-dependent quantiles yields valid class-conditional coverage, meaning the coverage guarantee holds separately within each class *ξ* ∈ Ξ.

### Flexible uncertainty calibration

As we shall see throughout the numerical experiments in “Results”, class-based CP provides a relatively modest improvement to uncertainty calibration over regular CP. While the step-function formulation provides a useful extension and yields class-conditional coverage, it is natural to admit even more flexible calibration functions. Specifically, we will allow the quantile to vary smoothly as a function of the input, thereby capturing finer-grained variations in the uncertainty structure across the input space.

This could be achieved by replacing the step function class in (9) with an appropriate class of smooth functions ${\mathscr{F}}$, leading to the following optimization problem:

10 $${\widehat{q}}_{\theta }:=\mathop{\arg \,\min }\limits_{{q}_{\theta }\in {\mathscr{F}}}\mathop{\sum }\limits_{i=1}^{{N}_{\mathrm{cal}}}{\ell }_{\alpha }\left({q}_{\theta }({{\bf{X}}}_{i}),{s}_{i}\right).$$

For example, ${\mathscr{F}}$ may be chosen as the Barron space^60^, which encompasses functions that can be efficiently approximated by shallow neural networks. In this case, the quantile function *q*_*θ*_ is parameterized with learnable parameters *θ*, allowing for input-dependent calibration that adapts continuously across the input domain.

Such an extension is a generalization of class partitioning of input space in “Class-based conformal prediction” to infinite dimensions. However, as shown in refs. ^61,62^, exact conditional coverage over an infinite-dimensional input space is infeasible, since the error bound in (4) scales with the number of partition classes. To mitigate this,^39^ introduces a surrogate calibration objective, yielding a relaxed but theoretically justified conditional guarantee. See SI Section 1.3 for more explanations. Apart from the above issue, we make the observation that the hyperparameter *α* is rather restrictive, which can only be chosen to bias under/overestimation. Although the coverage property entails from *α* is theoretically attractive, practically, one might prefer a general improvement of the quality of uncertainty instead of an exact coverage.

With this in mind, we propose a weighted objective functional that generalizes (10) and aligns the predicted uncertainty with the observed prediction error. Specifically, we define

11 $${\widehat{q}}_{\theta }=\mathop{\arg \,\min }\limits_{{q}_{\theta }\in {\mathscr{F}}}\mathop{\sum }\limits_{i=1}^{{N}_{\mathrm{cal}}}\widetilde{w}({{\bf{X}}}_{i},{{\bf{Y}}}_{i})\,\left|{q}_{\theta }({{\bf{X}}}_{i})-s({{\bf{X}}}_{i},{{\bf{Y}}}_{i})\right|,$$

where $\widetilde{w}({{\bf{X}}}_{i},{{\bf{Y}}}_{i})$ is a weight function. Note that when $\widetilde{w}$ is defined as

12 $$\widetilde{w}({{\bf{X}}}_{i},{{\bf{Y}}}_{i})=\left\{\begin{array}{ll}1-\alpha , & \,{\rm{if}}\,{q}_{\theta }({{\bf{X}}}_{i})-s({{\bf{X}}}_{i},{{\bf{Y}}}_{i})\ge 0,\\ \alpha , & \,{\rm{otherwise}}\,,\end{array}\right.$$

the minimization problem (11) reduces to (10). In practice, we use an equivalent formulation of (11)

13 $${\widehat{q}}_{\theta }=\mathop{\arg \,\min }\limits_{{q}_{\theta }\in {\mathscr{F}}}\mathop{\sum }\limits_{i=1}^{{N}_{\mathrm{cal}}}w({{\bf{X}}}_{i},{{\bf{Y}}}_{i})\,\left|{q}_{\theta }\,\sigma ({{\bf{X}}}_{i})-\parallel \widetilde{f}({{\bf{X}}}_{i})-{{\bf{Y}}}_{i}\parallel \right|,\,$$

where $\widetilde{w}({{\bf{X}}}_{i},{{\bf{Y}}}_{i})=w({{\bf{X}}}_{i},{{\bf{Y}}}_{i})/\sigma ({{\bf{X}}}_{i})$ so that the weights *w*(**X**_*i*_, **Y**_*i*_) can be chosen with the same unit as the error instead of the score, which is more intuitive. The weight is typically chosen to emphasize samples with larger observed errors.

This formulation prioritizes aligning predicted uncertainties with observed errors rather than enforcing theoretical coverage guarantees, thereby improving calibration quality in both overestimation and underestimation, as further demonstrated in “Results”. The overall method is illustrated in Fig. 1, and will be used consistently throughout our numerical experiments.

### Application to forces uncertainty

The framework introduced in the previous section is highly general and agnostic to the specific nature of the prediction task. In this section, we specialize the formulation to the context of machine-learned interatomic potentials (MLIPs), with a particular focus on the calibration of force uncertainties that are central to applications such as MD and active learning. Importantly, the same methodology can be readily extended to other atom-centred quantities (e.g., charges); however, calibrating global quantities (e.g., total energy) is more challenging due to the increased variance stemming from size-extensive effects and the typically limited size of available calibration datasets.

To begin with, consider an MLIP model trained to approximate the potential energy $E\in {\mathbb{R}}$ of a given atomic configuration ${\{{{\boldsymbol{r}}}_{i},{Z}_{i}\}}_{i=1}^{{N}_{{\rm{at}}}}$. If $\widetilde{E}\left({\{{{\boldsymbol{r}}}_{i}\}}_{i=1}^{{N}_{{\rm{at}}}}\right)$ is the predicted energy, then ${\widetilde{{\bf{F}}}}_{i}:=-{\nabla }_{{{\boldsymbol{r}}}_{i}}\widetilde{E}\in {{\mathbb{R}}}^{3}$ is the predicted force. For a *local* MLIP model, we can write

14 $${\widetilde{{\bf{F}}}}_{i}:=\widetilde{f}({{\bf{X}}}_{i}),$$

where **X**_*i*_ denotes the local atomic environment of atom *i*. For non-conservative force fields (14) is applied directly without reference to the MLIP energy. For future reference, we use ${\mathcal{X}}$ to denote the class of all atomic environments in a system of interest, so that the predicted atomic forces $\widetilde{f}:{\mathcal{X}}\to {{\mathbb{R}}}^{3}$. We assume that each prediction ${\widetilde{{\bf{F}}}}_{i}$ is accompanied by an associated uncertainty estimate ${\sigma }_{F}({{\bf{X}}}_{i})\in {{\mathbb{R}}}_{\, > \,0}$, derived from ensemble variance, dropout variance, posterior approximation (e.g., Laplace or variational inference), or other heuristic epistemic uncertainty estimators^13,18,54,55,63^. Table 3 summarizes representative MLIP architectures and their commonly used UQ approaches, which in this work provide the baseline uncertainty estimates *σ*_*F*_(**X**_*i*_). Although LLPR^40^ is used in this work as a representative baseline for deep equivariant models, it builds upon established concepts of prediction rigidity and D-optimality previously developed for linear machine-learning potentials^64,65^ and shallow neural networks^66,67^. These methods estimate uncertainty by analyzing the local density of training data in the descriptor space.

**Table 3:** Representative machine-learned interatomic potentials (MLIPs) and their commonly used uncertainty quantification (UQ) methods, with descriptions and references

MLIPs	Representative UQ	Descriptions	Foundation model	Refs.
ACE	Bayesian	Bayesian linear regression	No	^32^
DP	Ensemble	Model ensemble (different random seeds)	No	^22^
EquiformerV2	Feature distance	Distance in latent feature space	Yes	^70^
GAP	Bayesian	Gaussian process regression	No	^18^
HDNNP	Dropout	Monte Carlo dropout	No	^71^
MACE	Ensemble	Model ensemble (different random seeds)	No	^72^
MACE-MP-0	LLPR	Local linear prediction rigidities	Yes	^40^
MTP	D-optimality	D-optimality criterion in active learning	No	^50^
NEP	Ensemble	Model ensemble (different random seeds)	No	^73^
NequIP	Bayesian	Gaussian mixture posterior	No	^74^
SevenNet	Ensemble	Multi-head committee models	Yes	^75^
SNAP	POPS	Pointwise optimal parameter sets	No	^76^

To calibrate these per-atom force uncertainties, we define a site-specific conformal score function based on the pair (**X**_*i*_, **F**_*i*_), where ${{\bf{F}}}_{i}\in {{\mathbb{R}}}^{3}$ is the ground-truth DFT force on atom *i*:

15 $${s}_{F}({{\bf{X}}}_{i},{{\bf{F}}}_{i}):=\frac{\left\Vert {\widetilde{{\bf{F}}}}_{i}-{{\bf{F}}}_{i}\right\Vert }{{\sigma }_{F}({{\bf{X}}}_{i})}.$$

This formulation follows directly from the general conformal score in Eq. (2), applied at the atomic level by replacing the generic output **Y** with the per-atom force vector **F**_*i*_. The resulting score ${s}_{F}\in {{\mathbb{R}}}_{\ge 0}$ quantifies the normalized discrepancy between the predicted and true forces, scaled by the model’s heuristic uncertainty.

In the calibration framework, each atomic environment **X**_*i*_ is mapped to a descriptor vector, which serves as the input to the quantile model *q*_*θ*_. For traditional descriptors such as atom-centred symmetry functions^1^, smooth overlap of atomic positions^68^, or ACE^41,69^, these feature vectors are fixed functions of local geometry. In contrast, equivariant neural networks like MACE learn symmetry-adapted representations jointly with the potential, providing richer descriptors. In our case, we use a simple feed-forward neural network that maps trained MACE descriptors to quantile estimates. This demonstrates that the proposed calibration approach naturally accommodates both fixed and learned descriptors, and is therefore broadly applicable across MLIP architectures.

## Supplementary information

Supplementary Information

## Data Availability

Data and scripts supporting the findings of this study are available at 10.5281/zenodo.18709053. The MACE version and its LLPR implementation used throughout the article can be found in https://github.com/ACEsuit/mace/pull/601. The MACE foundation models used in this work can be found at https://github.com/ACEsuit/mace-foundations.

## Code availability

The MACE version and its LLPR implementation used throughout the article can be found in https://github.com/ACEsuit/mace/pull/601. The MACE foundation models used in this work can be found at https://github.com/ACEsuit/mace-foundations. Scripts for reproducing the plots will be made available upon publication.
