# Supplementary material for: Flexible uncertainty calibration for machine-learned interatomic potentials
Source: NPJ Comput Mater. 2026 Apr 27;12(1):225. doi: 10.1038/s41524-026-02080-3 (PMC13322957; doi:10.1038/s41524-026-02080-3)
Supplement: Supplementary file 1 — Supplementary Information [file 41524_2026_2080_MOESM1_ESM.pdf]

Supplementary Information for:

Flexible Uncertainty Calibration for Machine-Learned Interatomic Potentials

Cheuk Hin Ho[1], Christoph Ortner[1], Yangshuai Wang[2*]

[1]Department of Mathematics, University of British Columbia, Vancouver, V6T 1Z2, Canada.
[2]Department of Mathematics, National University of Singapore, 10 Lower Kent Ridge Road, 119076, Singapore.

*Corresponding author(s). E-mail(s): yswang@nus.edu.sg;

S1 Conformal Prediction Theory

This appendix presents additional theoretical foundations and technical details related to the conformal prediction framework, which are not fully discussed in the main text.

S1.1 Regular Conformal Prediction

We define a coverage function $\mathcal{C} : \mathcal{X} \rightrightarrows \mathcal{Y}$ as a *set-valued mapping*, meaning that for each input $\mathbf{X} \in \mathcal{X}$, the output $\mathcal{C}(\mathbf{X}) \subseteq \mathcal{Y}$ is a measurable subset of $\mathcal{Y}$ representing the predicted confidence region. Given a user-specified confidence level $\alpha \in (0, 1)$, the function $\mathcal{C}$ is constructed such that, for any test point $(\mathbf{X}_{\text{new}}, \mathbf{Y}_{\text{new}}) \sim P$, the following marginal coverage condition holds:

$$\mathbb{P}\big(\mathbf{Y}_{\text{new}} \in \mathcal{C}(\mathbf{X}_{\text{new}})\big) \geq 1 - \alpha. \tag{1}$$

Under finite-sample exchangeability, this condition can be more precisely bounded via the conformal calibration framework [1] as

$$1 - \alpha \leq \mathbb{P}\big(\mathbf{Y}_{\text{new}} \in \mathcal{C}(\mathbf{X}_{\text{new}})\big) \leq 1 - \alpha + \frac{1}{N_{\text{cal}} + 1},$$

ensuring that the coverage holds marginally over the randomness in $\mathbf{X}_{\text{new}}$ and $\mathcal{D}_{\text{cal}}$. The upper bound $1-\alpha+1/(N_{\text{cal}}+1)$ arises from the discrete nature of empirical quantile estimation under finite calibration size, and reflects the worst-case marginal coverage achievable under the exchangeability assumption.

Using Eq. 2, we compute calibration scores $\{s_i := s(\mathbf{X}_i, \mathbf{Y}_i)\}_{i=1}^{N_{\text{cal}}}$ for all entries in $\mathcal{D}_{\text{cal}}$. To enforce marginal coverage at confidence level $1-\alpha$, we compute the $(1-\alpha)$ empirical quantile $\hat{q} \in \mathbb{R}_{\geq 0}$ defined by

$$\hat{q} := \text{quantile}\left(\{s_i\}_{i=1}^{N_{\text{cal}}}, \frac{\lceil (N_{\text{cal}}+1)(1-\alpha) \rceil}{N_{\text{cal}}}\right).$$

This quantile formulation ensures that the conformal region satisfies the marginal coverage guarantee stated in Eq. (1) under the assumption of exchangeability.

The conformal prediction region at a new input $\mathbf{X}_{\text{new}}$ is then given by:

$$\mathcal{C}(\mathbf{X}_{\text{new}}) := \{\mathbf{Y} \in \mathcal{Y} \mid s(\mathbf{X}_{\text{new}}, \mathbf{Y}) \leq \hat{q}\}. \tag{2}$$

In other words, $\mathcal{C}(\mathbf{X}_{\text{new}})$ contains all plausible outputs $\mathbf{Y}$ that are within the $(1-\alpha)$ tolerance range defined by the quantile of calibration residuals.

S1.2 Class-based Conformal Prediction

To ensure valid conditional coverage within a class-based conformal prediction framework, we must rigorously define the associated coverage function, as motivated in Section S1.1 (cf. Eq. (2)).

Following the analytical framework in [2], and starting from Eq. (4) in the main text, we recall that the new conformity score $s_{\text{new}}(\mathbf{Y}) := s(\mathbf{X}_{\text{new}}, \mathbf{Y})$ is not compared to the $(1-\alpha)$ empirical quantile of the calibration scores $\{s_i\}_{i=1}^{N_{\text{cal}}}$, but instead to the adjusted quantile at level

$$\big(\lceil (N_{\text{cal}}+1)\cdot(1-\alpha) \rceil / N_{\text{cal}}\big).$$

This adjusted threshold arises from augmenting the calibration dataset with the unknown new score s_{new}.

To incorporate this perspective into quantile regression for conformity scores, we propose fitting a model $\hat{q}_{\text{cb}}$ on an augmented dataset that includes a guess s for the unobserved s_{new}. Formally, we define $\hat{q}_s$ as the minimizer of the empirical quantile loss:

$$\begin{aligned}\hat{q}_{\text{cb}}^{s} := \underset{q_{\text{cb}} \in \mathscr{F}_{\text{cb}}}{\arg\min} \frac{1}{N_{\text{cal}}+1} \sum_{i=1}^{N_{\text{cal}}} \ell_\alpha\big(q_{\text{cb}}(\mathbf{X}_i), s_i\big) \\ + \frac{1}{N_{\text{cal}}+1} \ell_\alpha\big(q_{\text{cb}}(\mathbf{X}_{\text{new}}), s\big),\end{aligned} \tag{3}$$

where $\mathscr{F}_{\text{cb}}$ denotes the class of quantile regression functions (detailed below), and $\ell_\alpha(\cdot,\cdot)$ is the standard asymmetric pinball loss associated with quantile level α.

The prediction set associated with input $\mathbf{X}_{\text{new}}$ is then defined as

$$\mathcal{C}(\mathbf{X}_{\text{new}}) := \{\mathbf{Y} \in \mathcal{Y} : s(\mathbf{X}_{\text{new}}, \mathbf{Y}) \leq \hat{q}_{\text{cb}}^{s_{\text{new}}}(\mathbf{X}_{\text{new}})\} . \tag{4}$$

The function class $\mathscr{F}_{\text{cb}}$ is chosen to be the space of classwise constant (i.e., step) functions defined over a finite partition Ξ of the covariate space:

$$\mathscr{F}_{\text{cb}} := \left\{ x \mapsto \sum_{\xi \in \Xi} q_\xi \cdot \mathbf{1}\{x \in \xi\} : q_\xi \in \mathbb{R}, \forall \xi \in \Xi \right\},$$

where $\mathbf{1}\{\cdot\}$ denotes the indicator function.

Under this construction, it is shown in [2, Corollary 1] that the prediction set $\mathcal{C}(\mathbf{X}_{\text{new}})$ satisfies the following conditional coverage guarantee for any class $\xi \in \Xi$:

$$\mathbb{P}\left(\mathbf{Y}_{\text{new}} \in \mathcal{C}(\mathbf{X}_{\text{new}}) \,|\, \mathbf{X}_{\text{new}} \in \xi\right) \geq 1 - \alpha.$$

Moreover, if the conditional distribution of the conformity score $s \mid \mathbf{X}$ is continuous, then the coverage error admits the following upper bound:

$$\begin{aligned} &\mathbb{P}\left(\mathbf{Y}_{\text{new}} \in \mathcal{C}(\mathbf{X}_{\text{new}}) \,|\, \mathbf{X}_{\text{new}} \in \xi\right) \\ &\quad \leq 1 - \alpha + \frac{|\Xi|}{(N_{\text{cal}} + 1) \cdot \mathbb{P}(\mathbf{X}_{\text{new}} \in \xi)}. \end{aligned} \tag{5}$$

This bound explicitly characterizes the effect of class granularity and calibration set size on the worst-case conditional coverage deviation.

S1.3 Flexible Uncertainty Calibration

For flexible uncertainty calibration, the goal is to construct valid prediction sets without restricting to a finite-dimensional function class. However, prior work [3, 4] shows that exact conditional coverage is unattainable in general infinite-dimensional settings. We therefore seek relaxed guarantees under suitable assumptions, analogous to the relaxation introduced earlier.

To this end, note that direct optimization over an unrestricted function class is intractable. To address this issue and ensure well-posedness of the quantile regression problem, we introduce regularization. Specifically, for a chosen regularization functional $\mathcal{R}(\cdot)$, we define:

$$\begin{aligned} \hat{q}_\theta^s := \arg\min_{q_\theta \in \mathscr{F}} \Bigg(&\frac{1}{N_{\text{cal}} + 1} \sum_{i=1}^{N_{\text{cal}}} \ell_\alpha\left(q_\theta(\mathbf{X}_i), s_i\right) \\ &+ \frac{1}{N_{\text{cal}} + 1} \ell_\alpha\left(q_\theta(\mathbf{X}_{\text{new}}), s\right) + \mathcal{R}(q_\theta) \Bigg) . \end{aligned} \tag{6}$$

Here, the regularization term $\mathcal{R}(q_\theta)$ promotes desirable properties such as smoothness or sparsity, and serves to control the effective complexity of the function class $\mathscr{F}$.

Having obtained $\hat{q}_\theta^s$, we define the prediction set as:

$$\mathcal{C}(\mathbf{X}_{\text{new}}) := \left\{\mathbf{Y} \in \mathcal{Y} : s(\mathbf{X}_{\text{new}}, \mathbf{Y}) \leq \hat{q}_\theta^{s_{\text{new}}}(\mathbf{X}_{\text{new}})\right\}.$$

Under mild regularity conditions on $\mathcal{R}(\cdot)$, a relaxed conditional coverage guarantee can be established, as formalized in [2, Theorem 3]. In particular, the deviation from the target coverage level $1-\alpha$ is shown to depend on the smoothness and capacity control induced by the regularizer.

We further note that flexible uncertainty calibration can naturally incorporate weighted loss formulations (cf. Eq. 11), which may be viewed as inducing an implicit regularization on the quantile estimator. This extension suggests the possibility of relaxed coverage guarantees, although a formal statistical analysis is left for future work.

S1.4 Bayesian Interpretation

The connection between the proposed calibration scheme and the Bayesian framework can be interpreted through the lens of uncertainty quantification in predictive distributions. In particular, we may view the heuristic uncertainty $\sigma(\mathbf{X})$ used in conformal calibration as an input-dependent scaling of the predictive variance under a Bayesian posterior.

Let $\Theta \in \mathbb{R}^p$ denote the neural network weights, and let $\mathcal{D}_{\text{train}}$ denote the training dataset. A common approximation in Bayesian neural networks is to assume a Gaussian posterior over parameters via Laplace approximation around the maximum a posteriori (MAP) estimate Θ_0:

$$p(\Theta \mid \mathcal{D}_{\text{train}}) \approx \mathcal{N}(\Theta_0, \mathbf{\Sigma}), \tag{7}$$

where $\mathbf{\Sigma}$ is the inverse Hessian of the negative log-posterior evaluated at Θ_0, i.e.,

$$\mathbf{\Sigma}^{-1} \approx \nabla_\Theta^2 \left[-\log p(\mathcal{D}_{\text{train}} \mid \Theta) - \log p(\Theta)\right]_{\Theta=\Theta_0}.$$

Under this posterior approximation, the predictive distribution at a new input $\mathbf{X}^*$ is given by:

$$p(\mathbf{Y} \mid \mathbf{X}^*, \mathcal{D}_{\text{train}}) \approx \mathcal{N}\left(\hat{f}_{\Theta_0}(\mathbf{X}^*),\ J(\mathbf{X}^*)\mathbf{\Sigma}J(\mathbf{X}^*)^\top\right), \tag{8}$$

where $J(\mathbf{X}^*) = \nabla_\Theta \hat{f}_\Theta(\mathbf{X}^*) \in \mathbb{R}^{d_y \times p}$ is the Jacobian of the model output with respect to the parameters, evaluated at Θ_0.

In our framework, we do not explicitly compute $\mathbf{\Sigma}$ or $J(\mathbf{X}^*)$; instead, we apply conformal calibration using a scalar uncertainty proxy $\sigma(\mathbf{X}^*)$. This can be interpreted as approximating the predictive variance by a scalar-scaled version of the global posterior covariance:

$$p(\mathbf{Y} \mid \mathbf{X}^*, \mathcal{D}_{\text{train}}) \approx \mathcal{N}\left(\hat{f}_{\Theta_0}(\mathbf{X}^*),\ \alpha^2(\mathbf{X}^*)\mathbf{\Sigma}\right), \tag{9}$$

where $\alpha(\mathbf{X}^*)$ is a data-dependent scaling factor learned via conformal calibration. The role of $\alpha(\mathbf{X}^*)$ is to adjust the (possibly misspecified) model-derived uncertainty to achieve correct frequentist coverage.

Conformal prediction can be interpreted as a nonparametric correction to a misspecified Bayesian posterior, where $\alpha(\mathbf{X}^*)$ accounts for local variability not captured by the global covariance $\mathbf{\Sigma}$. This guarantees valid uncertainty quantification regardless of the quality of the base estimate $\sigma(\mathbf{X})$, whether obtained from dropout, ensembles, or other approximations.

S2 MACE Architecture

MACE [5] is an E(3)-equivariant message passing neural network (EMPNN) model that uses higher-body-order messages. The expressiveness of the model is improved by using efficient multi-body messages instead of two-body messages. Multi-body messages reduce the number of network layers required to achieve the same expressiveness under two-body messages, resulting in a fast and highly parallelizable model. The MACE architecture follows the general framework of MPNNs and includes three parts: message construction, update, and readout.

Message passing: In message construction, MACE combines equivariant message passing with efficient many-body messages. The edges are embedded using a learnable radial basis $R^{(t)}_{kl_1l_2l_3}$ and a set of spherical harmonics $Y^{m_1}_{l_1}$, and the self-interaction is performed on the features $h^{(t)}_{j,\tilde{k}l_2m_2}$ with learnable weights $W^{(t)}_{\tilde{k}kl_2}$. $\boldsymbol{A}^{(t)}_{i,kl_3m_3}$ is the two-body feature obtained by pooling neighbor atoms:

$$A^{(t)}_{i,kl_3m_3} = \sum_{l_1m_1,l_2m_2} C^{l_3m_3}_{l_1m_1,l_2m_2} \sum_{j\in\mathcal{N}(i)} R^{(t)}_{kl_1l_2l_3}(r_{ji}) \cdot \cdot Y^{m_1}_{l_1}(\hat{r}_{ji}) \sum_{\tilde{k}} W^{(t)}_{\tilde{k}kl_2} h^{(t)}_{j,\tilde{k}l_2m_2}, \tag{10}$$

where $C^{l_3m_3}_{l_1m_1,l_2m_2}$ are the standard Clebsch–Gordan coefficients [6]. The key operation of MACE is to construct a multi-body feature $\boldsymbol{B}^{(t)}_{i,\eta_\nu kLM}$ through the tensor product of the two-body features $A^{(t)}_{i,kl_3m_3}$:

$$B^{(t)}_{i,\eta_\nu kLM} = \sum_{lm} C^{LM}_{\eta_\nu,lm} \prod_{\xi=1}^{\nu} \sum_{\tilde{k}} w^{(t)}_{\tilde{k}kl_\xi} A^{(t)}_{i,\tilde{k}l_\xi m_\xi}, \tag{11}$$

where the coupling coefficients $C^{LM}_{\eta_\nu}$ correspond to the generalized Clebsch–Gordan coefficients and $w^{(t)}_{\tilde{k}kl_\xi}$ is the learnable weight for the self-interaction of $A^{(t)}_{i,kl_3m_3}$. Finally, the message $m^{(t)}_i$ can be written as a linear expansion:

$$m^{(t)}_{i,kLM} = \sum_{\nu} \sum_{\eta_\nu} W^{(t)}_{z_i;kL,\eta_\nu} B^{(t)}_{i,\eta_\nu kLM}. \tag{12}$$

Update: The update is a linear function of the message and the residual connection [7]:

$$\begin{aligned} h_{i,kLM}^{(t+1)} &= U_t^k(\sigma_i^{(t)}, m_i^{(t)}) \\ &= \sum_{\tilde{k}} W_{kL,\tilde{k}}^{(t)} m_{i,\tilde{k}LM}^{(t)} + \sum_{\tilde{k}} W_{z_i,\tilde{k}}^{(t)} h_{i,\tilde{k}LM}^{(t)}. \end{aligned} \tag{13}$$

Readout: The readout is a mapping from the invariant part of the node features to a hierarchical decomposition of site energies:

$$E_i = E_i^{(0)} + E_i^{(1)} + \cdots + E_i^{(T)}, \tag{14}$$

where

$$E_i^{(t)} = \mathcal{R}_t(h_i^{(t)}) = \begin{cases} \sum_{\tilde{k}} W_{\text{readout},\tilde{k}}^{(t)} h_{i,\tilde{k}00}^{(t)}, & t < T, \\ \text{MLP}_{\text{readout}}^{(t)} \left(\{ h_{i,k00}^{(t)} \}_k \right), & t = T. \end{cases} \tag{19}$$

The readout only depends on the invariant features $h_{i,k00}^{(t)}$, making the site energy contribution $E_i^{(t)}$ invariant.

S3 LLPR Uncertainty Quantification

This section introduces the LLPR uncertainty quantification method [8], which defines prediction rigidities via a constrained optimization formulation. We consider a standard regression task with training data $\mathcal{D} = \{(\mathbf{x}_i, y_i)\}_{i=1}^{N_{\text{train}}}$, where $\mathbf{x}_i \in \mathbb{R}^d$ are input features and $y_i \in \mathbb{R}$ are scalar targets. The model prediction is denoted by $\tilde{y}(\mathbf{x}_i, \mathbf{w})$, and the empirical loss is given by

$$\mathcal{L}(\mathbf{w}) = \sum_{i=1}^{N_{\text{train}}} \ell\big(\tilde{y}(\mathbf{x}_i, \mathbf{w}), y_i\big).$$

The concept of prediction rigidity is based on evaluating how sensitive a model prediction $\tilde{y}(\mathbf{x}_i, \mathbf{w})$ is to perturbations away from its optimal value $\tilde{y}(\mathbf{x}_\star, \mathbf{w}_\circ)$, where $\mathbf{w}_\circ$ denotes the optimized model parameters [9]. To quantify this sensitivity for a specific test point $\mathbf{x}_\star$, a modified loss function $\mathcal{L}_c$ is introduced by adding a Lagrange multiplier term that enforces a constraint on the predicted value $\tilde{y}(\mathbf{x}_\star, \mathbf{w})$, forcing it to take an arbitrary value $\epsilon_\star$:

$$\mathcal{L}_c(\mathbf{w}, \lambda, \epsilon_\star) = \mathcal{L}(\mathbf{w}) + \lambda\big(\epsilon_\star - \tilde{y}(\mathbf{x}_\star, \mathbf{w})\big). \tag{15}$$

After solving the constrained optimization problem $\partial\mathcal{L}_c/\partial\mathbf{w} = 0$ and $\partial\mathcal{L}_c/\partial\lambda = 0$, the prediction rigidity of $\tilde{y}(\mathbf{x}_\star, \mathbf{w}_\circ)$ is defined as

$$R_\star = \left. \frac{\partial^2 \mathcal{L}_c(\epsilon_\star)}{\partial \epsilon_\star^2} \right|_{\epsilon_\star = \tilde{y}(\mathbf{x}_\star, \mathbf{w}_\circ)}.$$

This quantity reflects the sensitivity of the minimized loss to perturbations in the predicted value $\tilde{y}(\mathbf{x}_\star, \mathbf{w}_\circ)$. A larger $R_\star$ implies higher model confidence in the prediction. To compute this analytically, we apply a second-order approximation of the loss around the optimum $\mathbf{w}_\circ$:

$$\mathcal{L}(\mathbf{w}) \approx \mathcal{L}(\mathbf{w}_\circ) + \frac{1}{2}(\mathbf{w} - \mathbf{w}_\circ)^\top \mathbf{H}_\circ (\mathbf{w} - \mathbf{w}_\circ),$$

where the linear term vanishes due to the first-order optimality condition. Linearizing the prediction near $\mathbf{w}_\circ$, the constrained loss becomes [8, Section 3.2]

$$\mathcal{L}_c(\epsilon_\star) \approx \mathcal{L}(\mathbf{w}_\circ) + \frac{1}{2} \frac{(\epsilon_\star - \tilde{y}(\mathbf{x}_\star, \mathbf{w}_\circ))^2}{\left.\frac{\partial \tilde{y}_\star}{\partial \mathbf{w}}\right|_{\mathbf{w}_\circ}^\top \mathbf{H}_\circ^{-1} \left.\frac{\partial \tilde{y}_\star}{\partial \mathbf{w}}\right|_{\mathbf{w}_\circ}}.$$

The rigidity $R_\star$ then has the closed-form:

$$R_\star = \left(\left.\frac{\partial \tilde{y}_\star}{\partial \mathbf{w}}\right|_{\mathbf{w}_\circ}^\top \mathbf{H}_\circ^{-1} \left.\frac{\partial \tilde{y}_\star}{\partial \mathbf{w}}\right|_{\mathbf{w}_\circ} \right)^{-1}.$$

The Hessian $\mathbf{H}_\circ$ can be precomputed from the training set. While computing the exact Hessian is expensive due to second-order derivatives, a practical and widely used approximation is the generalized Gauss–Newton form:

$$\begin{aligned}
\mathbf{H}_\circ &= \frac{\partial^2 \mathcal{L}}{\partial \mathbf{w} \partial \mathbf{w}^\top} = \frac{\partial}{\partial \mathbf{w}} \sum_i \frac{\partial \ell_i}{\partial \tilde{y}_i} \frac{\partial \tilde{y}_i}{\partial \mathbf{w}^\top} \\
&= \sum_i \left(\frac{\partial \ell_i}{\partial \tilde{y}_i} \frac{\partial^2 \tilde{y}_i}{\partial \mathbf{w} \partial \mathbf{w}^\top} + \frac{\partial \tilde{y}_i}{\partial \mathbf{w}} \frac{\partial^2 \ell_i}{\partial \tilde{y}_i^2} \frac{\partial \tilde{y}_i}{\partial \mathbf{w}^\top} \right) \\
&\approx \sum_i \frac{\partial \tilde{y}_i}{\partial \mathbf{w}} \frac{\partial^2 \ell_i}{\partial \tilde{y}_i^2} \frac{\partial \tilde{y}_i}{\partial \mathbf{w}^\top},
\end{aligned} \tag{16}$$

where all derivatives are evaluated at $\mathbf{w}_\circ$. The approximation neglects the first term under the assumption that the model is well-trained ($\partial \ell_i / \partial \tilde{y}_i \approx 0$). This form is commonly used in optimization and UQ [10–13].

S4 Supplementary Numerical Experiments

S4.1 Spearman Rank Correlation Coefficient

The Spearman rank correlation coefficient (ρ) is a nonparametric statistic that measures the strength and direction of a monotonic relationship between two variables. Unlike the Pearson coefficient, which evaluates linear dependence, Spearman correlation is based solely on ranked values. In the context of uncertainty quantification,

ρ provides a direct assessment of whether larger predicted uncertainties are consistently associated with larger observed errors, thereby serving as a standard metric for evaluating the quality of uncertainty ranking.

Given two sets of observations, predicted uncertainties $\{u_i\}_{i=1}^N$ and observed errors $\{e_i\}_{i=1}^N$, we assign ranks $R(u_i)$ and $R(e_i)$ to each value. The coefficient ρ is then defined as the Pearson correlation between these ranks:

$$\rho = \frac{\sum_{i=1}^N \left(R(u_i) - \overline{R(u)}\right)\left(R(e_i) - \overline{R(e)}\right)}{\sqrt{\sum_{i=1}^N \left(R(u_i) - \overline{R(u)}\right)^2}\sqrt{\sum_{i=1}^N \left(R(e_i) - \overline{R(e)}\right)^2}}. \tag{17}$$

In the absence of ties, ρ admits closed form

$$\rho = 1 - \frac{6\sum_{i=1}^N d_i^2}{N(N^2-1)}, \tag{18}$$

where $d_i = R(u_i) - R(e_i)$ is the rank difference for each observation.

A value of $\rho = 1$ corresponds to perfect agreement between uncertainty ranking and error ranking, $\rho = -1$ indicates perfect disagreement, and $\rho = 0$ signifies no monotonic association. In practice, higher ρ values indicate better alignment between predicted uncertainties and true errors, and are thus desirable for evaluating the effectiveness of calibration schemes.

S4.2 Flexible UC on Other Public Datasets

We further evaluate the proposed calibration scheme on two widely used public datasets, MPtraj and MATPES, with results shown in Figures 1 and 2. These datasets are representative benchmarks for molecular and materials modeling and are often used for training and validation of foundation MLIPs. In this context, calibration provides a useful test of whether the proposed framework delivers consistent benefits beyond the custom benchmarks considered in the main text.

In both MPtraj and MATPES, the improvements achieved by flexible uncertainty calibration are less pronounced compared with the LiCl and catalytic surface benchmarks discussed in the main text. This outcome is expected, since the distributional gap between calibration and testing sets within MPtraj, or between MPtraj and MATPES, is relatively small. As a result, both qualitative improvements in the error–uncertainty correlation and quantitative gains in rank-based metrics remain modest. Put differently, when training and testing environments are already closely matched, the baseline LLPR and regular CP strategies provide reasonably reliable estimates, leaving less room for additional gains from more flexible calibration.

Nevertheless, the experiments on MPtraj and MATPES provide two important insights. First, they confirm that flexible calibration does not deteriorate performance even in low-shift regimes, thereby maintaining consistency with standard approaches. Second, they highlight that the advantages of flexible UC are most significant when substantial distributional shifts are present, such as in ionic systems or heterogeneous

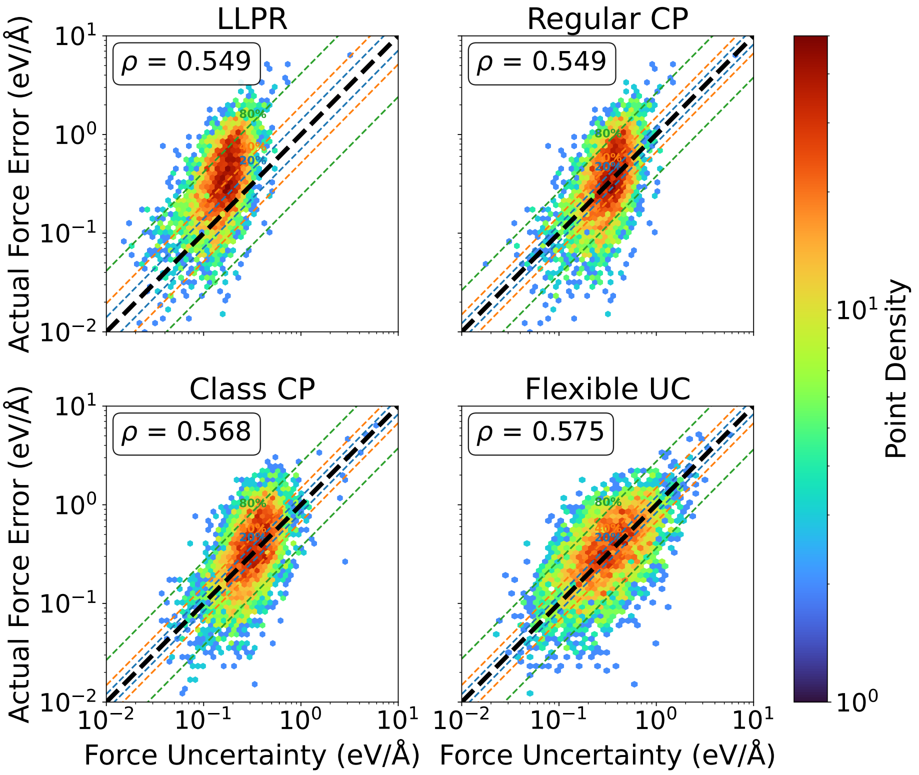

Fig. 1: Uncertainty estimates from LLPR, regular CP, class-based CP, and flexible UC on the MATPES dataset.

catalytic environments. In practice, this suggests that flexible UC may be especially valuable in active learning and transfer settings, where models are repeatedly exposed to configurations far from the calibration distribution.

Taken together, these results demonstrate that flexible UC provides a general and robust framework: it delivers substantial improvements when extrapolation is required, while ensuring comparable performance to existing methods when calibration and testing domains are similar.

S4.3 Effect of different α

Figure 3 shows predicted versus actual force errors for regular and class-conditional CP across several α values. Each panel displays the joint distribution of predicted and realized errors, with the dashed black line indicating the ideal 1:1 correspondence.

Crucially, for all values of α, the ranking quality remains limited. Class-conditional CP exhibits a slightly higher Spearman rank correlation ($\rho \approx 0.35$) than regular CP

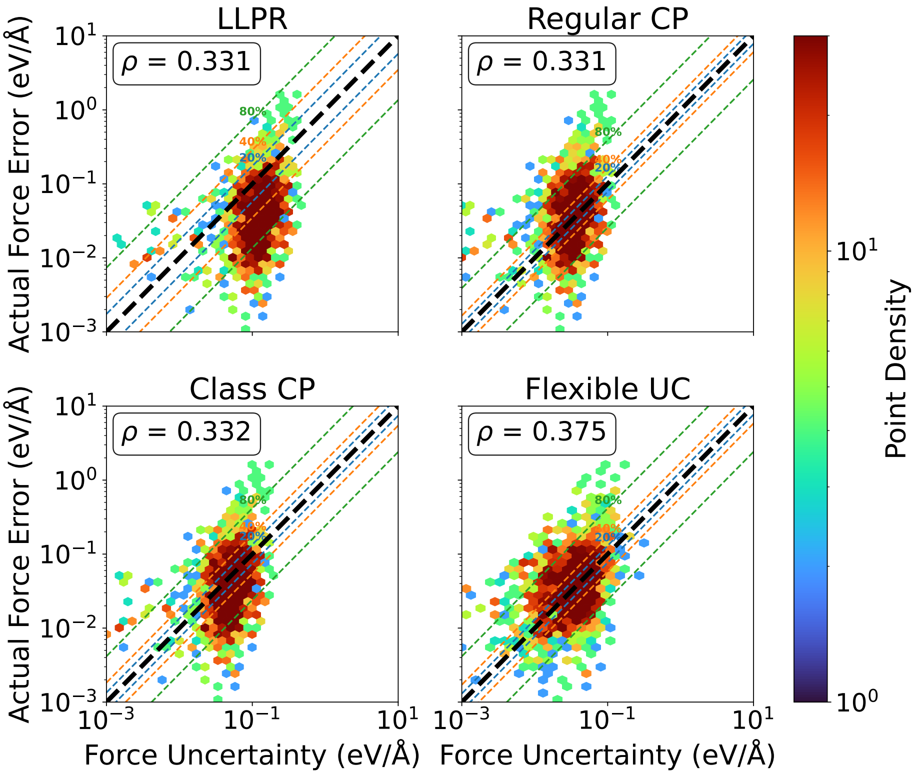

Fig. 2: Uncertainty estimates from LLPR, regular CP, class-based CP, and flexible UC on the MPtraj dataset.

($\rho \approx 0.31$), but these values are largely invariant to the choice of α. The overall shape of the distributions remains qualitatively similar, shifting only in scale.

This observation highlights a fundamental limitation of standard CP: simply varying α acts as a global rescaling. It is insufficient to capture the complex, heterogeneous uncertainty landscape. This finding further motivates the need for Flexible UC, which goes beyond global rescaling to learn a local, environment-dependent calibration.

S4.4 Model-agnostic nature of Flexible UC

To demonstrate the model-agnostic nature of Flexible UC, we replicate the calibration workflow using an uncertainty baseline distinct from the LLPR approach employed in the main text. Specifically, we consider an ensemble (committee) uncertainty estimator constructed from three distinct versions of the MACE foundation model (`mp0b`, `mp0b2`, and `mp0b3`).

Figure 4 compares the relationship between the predicted force uncertainty and the corresponding force error for this ensemble-based baseline on the LiCl dataset.

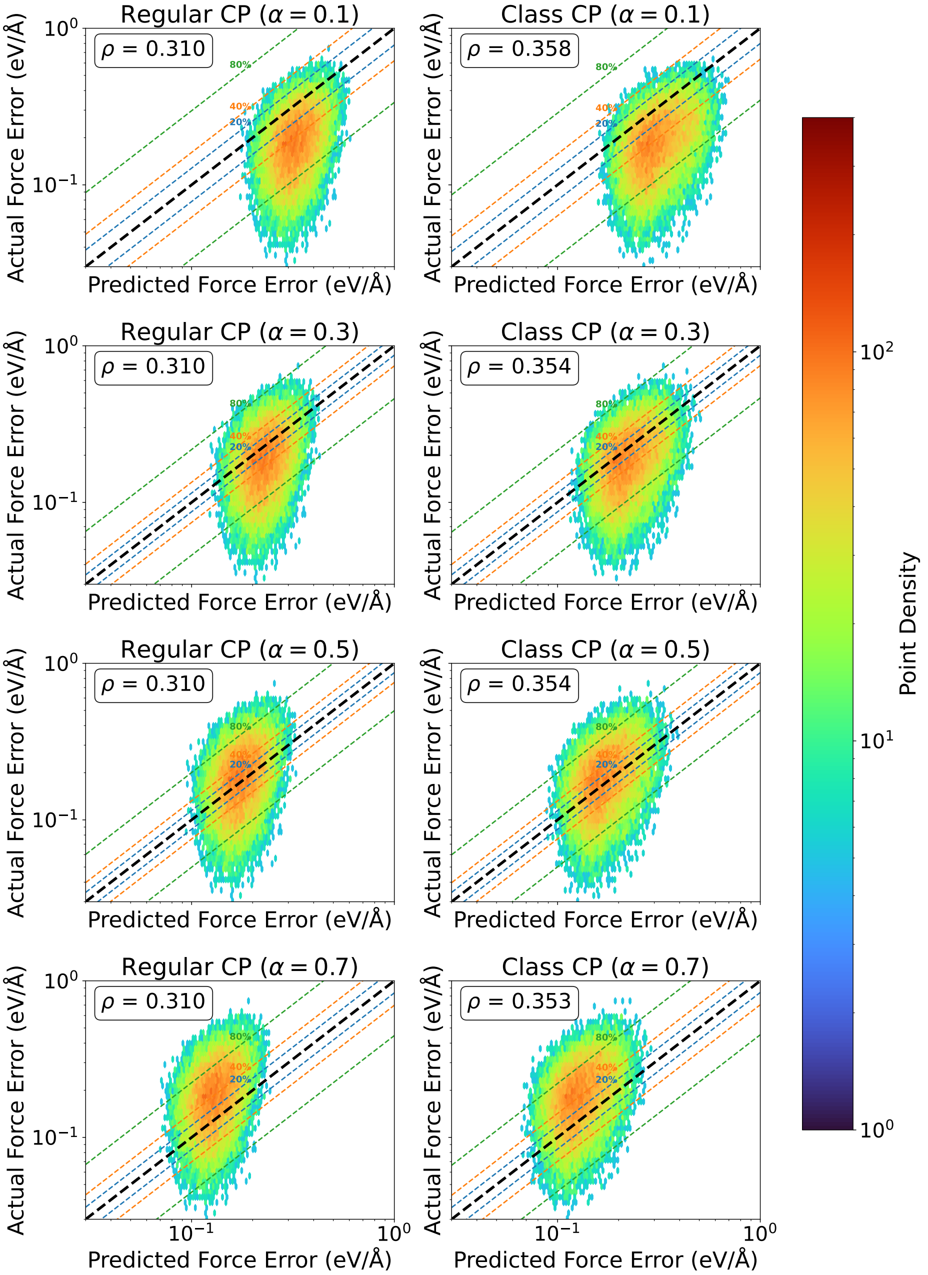

Fig. 3: Comparison of regular CP and class-conditional CP uncertainty estimates on the LiCl dataset for different miscoverage levels α. The Spearman rank correlation coefficient ρ quantifies the monotonic association between predicted uncertainty and force error.

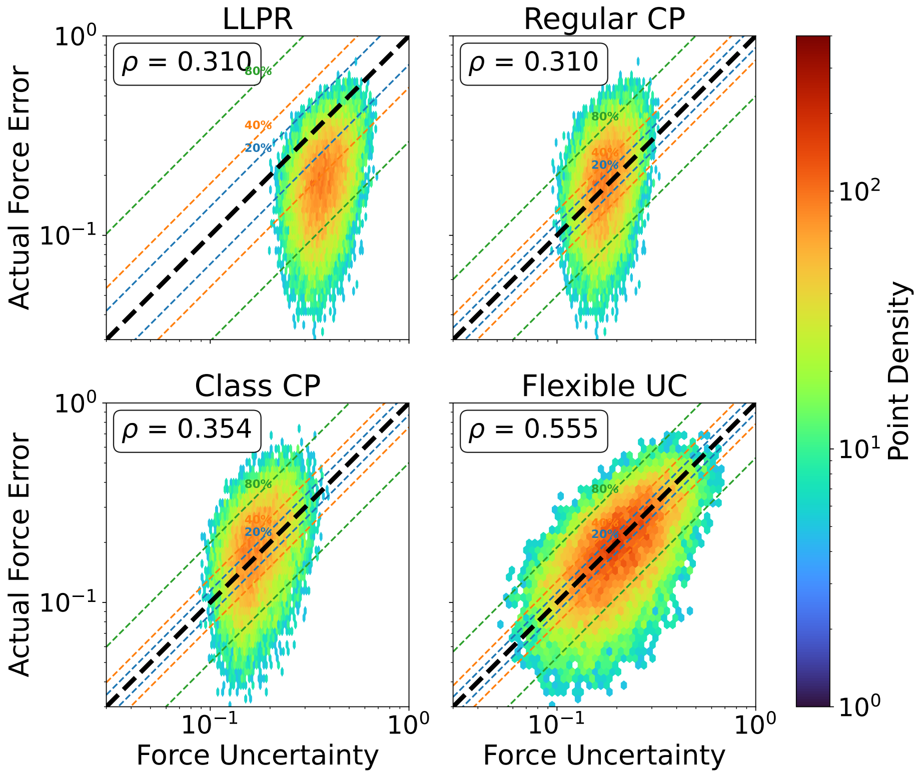

Fig. 4: Calibration performance on the LiCl dataset using an ensemble-based uncertainty baseline. Comparison includes raw ensemble uncertainty, regular CP, class-conditional CP, and Flexible UC. ρ denotes the Spearman rank correlation coefficient between uncertainty and force error. All force units are reported in eV/Å.

The committee-based ensemble exhibits a slightly weaker rank correlation between predicted uncertainty and force error ($\rho = 0.310$) compared to the LLPR baseline ($\rho = 0.386$). Regular CP provides no improvement in ranking ($\rho = 0.310$), while Class CP yields a modest gain ($\rho = 0.360$). It is worth noting that, in contrast to LLPR which tends to underestimate errors, the ensemble-based uncertainties in this setting systematically overestimate the true force error.

Despite this difference in baseline behavior, the uncertainties calibrated using Flexible UC show a substantially stronger monotonic relationship with the true force error, achieving a Spearman rank correlation of $\rho = 0.555$. This result is quantitatively consistent with the performance achieved using LLPR ($\rho = 0.589$).

These findings confirm that Flexible UC operates effectively as a post-processing step without modifying the underlying model architecture. Whether the baseline uncertainty is derived from local rigidities (LLPR) or model committees (Ensembles), and whether it tends to under- or over-estimate risk, Flexible UC exploits the empirical

error-uncertainty relationship to deliver robust and consistent calibration. This provides direct empirical evidence supporting the general applicability of the framework across different UQ paradigms.

S4.5 Illustration of the Weighted Loss Function

To penalize high-error configurations more strongly, we introduce a weighted loss in which each data point $(\mathbf{X}_i, \mathbf{Y}_i)$ is assigned a weight w depending on its prediction error. Formally,

$$w := w_{c_0}(\epsilon) = \text{sigmoid}\big(c_0(\epsilon - 0.05)\big) + 0.3, \tag{19}$$

where $\epsilon := \|\tilde{f}(\mathbf{X}_i) - \mathbf{Y}_i\|$ denotes the prediction error and c_0 is a tunable hyperparameter. This design smoothly increases w as the error grows, thereby emphasizing configurations with larger deviations.

Figure 5 shows the dependence of $w_{c_0}(\epsilon)$ on ϵ across different c_0 values. In all experiments we set $c_0 = 40$, and we found that moderate changes of this parameter do not significantly affect the results.

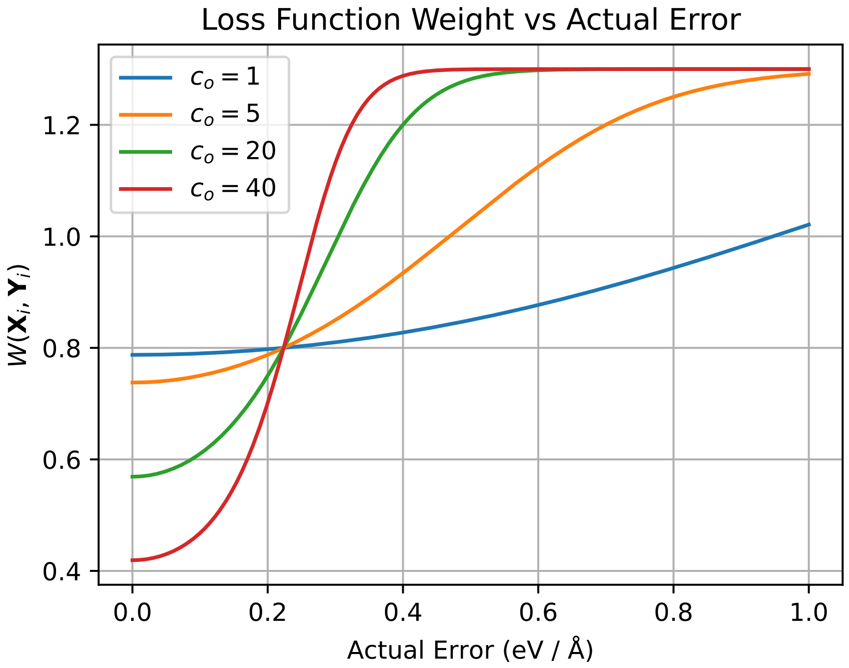

Fig. 5: Weighted loss function $w_{c_0}(\epsilon)$ for different choices of c_0.

S4.6 Descriptor-based Quantile Model and Implementation Details

We briefly outline the implementation details of flexible UC and class-based CP. For flexible UC, we adopt a deliberately simple design for the quantile model. A feedforward neural network learns a multiplicative adjustment $q(\mathbf{X})$ to the baseline uncertainty estimate σ, based on MACE descriptors $h(\mathbf{X}) \in \mathbb{R}^d$. The model computes

$$q(\mathbf{X}) = \sigma \cdot \mathrm{Softplus}(f_\theta(h(\mathbf{X}))), \tag{20}$$

where

$$f_\theta(h(\mathbf{X})) = W_3\, \phi(W_2\, \phi(W_1 h(\mathbf{X}) + b_1) + b_2) + b_3, \tag{21}$$

with $\phi(\cdot)$ denoting the ReLU activation. The hidden dimension of each dense layer is set to 64, except for the final scalar output prior to the Softplus, which ensures positivity of the predicted scaling factor. All quantile models are trained using the Adam optimizer with learning rate 10^{-3}, implemented in `PyTorch`.

For class-based CP, we set the number of classes to $N_{\mathrm{class}} = 20$ and partition local atomic environments using the `BayesianGaussianMixture` implementation in `scikit-learn`. In datasets with highly diverse environments (e.g., MATPES, MPtraj, OMOL), the number of classes is empirically increased until a clear separation of score distributions is achieved; further refinement beyond this point yields negligible changes.

References

[1] Romano, Y., Patterson, E. & Candes, E. Conformalized quantile regression. *Advances in neural information processing systems* **32** (2019).

[2] Gibbs, I., Cherian, J. J. & Candès, E. J. Conformal prediction with conditional guarantees. *Journal of the Royal Statistical Society Series B: Statistical Methodology* qkaf008 (2025).

[3] Foygel Barber, R., Candes, E. J., Ramdas, A. & Tibshirani, R. J. The limits of distribution-free conditional predictive inference. *Information and Inference: A Journal of the IMA* **10**, 455–482 (2021).

[4] Vovk, V. *Conditional validity of inductive conformal predictors*, 475–490 (PMLR, 2012).

[5] Batatia, I., Kovacs, D. P., Simm, G., Ortner, C. & Csányi, G. Mace: Higher order equivariant message passing neural networks for fast and accurate force fields. *Advances in Neural Information Processing Systems* **35**, 11423–11436 (2022).

[6] de Swart, J. J. in *The octet model and its clebsch-gordan coefficients* 120–143 (CRC Press, 2018).

[7] He, K., Zhang, X., Ren, S. & Sun, J. *Deep residual learning for image recognition*, 770–778 (2016).

[8] Bigi, F., Chong, S., Ceriotti, M. & Grasselli, F. A prediction rigidity formalism for low-cost uncertainties in trained neural networks. *Machine Learning: Science and Technology* **5**, 045018 (2024).

[9] Chong, S. *et al.* Robustness of local predictions in atomistic machine learning models. *Journal of Chemical Theory and Computation* **19**, 8020–8031 (2023).

[10] Schraudolph, N. N. Fast curvature matrix-vector products for second-order gradient descent. *Neural computation* **14**, 1723–1738 (2002).

[11] Holzmüller, D., Zaverkin, V., Kästner, J. & Steinwart, I. A framework and benchmark for deep batch active learning for regression. *Journal of Machine Learning Research* **24**, 1–81 (2023).

[12] Levenberg, K. A method for the solution of certain non-linear problems in least squares. *Quarterly of applied mathematics* **2**, 164–168 (1944).

[13] Marquardt, D. W. An algorithm for least-squares estimation of nonlinear parameters. *Journal of the society for Industrial and Applied Mathematics* **11**, 431–441 (1963).
